# Narrow Pressure Stability Window of Gas Diffusion Electrodes Limits the Scale-Up of CO_2_ Electrolyzers

**DOI:** 10.1021/acssuschemeng.2c00195

**Published:** 2022-03-29

**Authors:** Lorenz M. Baumgartner, Christel I. Koopman, Antoni Forner-Cuenca, David A. Vermaas

**Affiliations:** † Department of Chemical Engineering, Delft University of Technology, Van der Maasweg 9, 2629 HZ Delft, Netherlands; ‡ Department of Chemical Engineering and Chemistry, Eindhoven University of Technology, Het Kranenveld 14, 5612 AZ Eindhoven, Netherlands

**Keywords:** CO_2_ reduction, electrochemistry, electrochemical engineering, gas diffusion electrode, scale-up

## Abstract

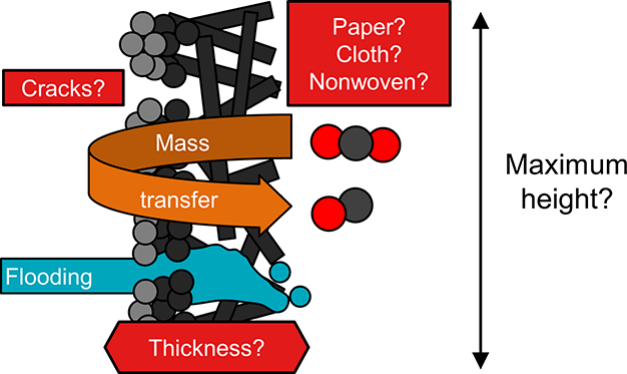

Electrochemical CO_2_ reduction is a promising process to store intermittent renewable energy in the form of chemical bonds and to meet the demand for hydrocarbon chemicals without relying on fossil fuels. Researchers in the field have used gas diffusion electrodes (GDEs) to supply CO_2_ to the catalyst layer from the gas phase. This approach allows us to bypass mass transfer limitations imposed by the limited solubility and diffusion of CO_2_ in the liquid phase at a laboratory scale. However, at a larger scale, pressure differences across the porous gas diffusion layer can occur. This can lead to flooding and electrolyte breakthrough, which can decrease performance. The aim of this study is to understand the effects of the GDE structure on flooding behavior and CO_2_ reduction performance. We approach the problem by preparing GDEs from commercial substrates with a range of structural parameters (carbon fiber structure, thickness, and cracks). We then determined the liquid breakthrough pressure and measured the Faradaic efficiency for CO at an industrially relevant current density. We found that there is a trade-off between flooding resistance and mass transfer capabilities that limits the maximum GDE height of a flow-by electrolyzer. This trade-off depends strongly on the thickness and the structure of the carbon fiber substrate. We propose a design strategy for a hierarchically structured GDE, which might offer a pathway to an industrial scale by avoiding the trade-off between flooding resistance and CO_2_ reduction performance.

## Abstract 2

Commonly used gas diffusion electrodes for electrochemical CO_2_ reduction suffer from poor scalability due to their limited flooding resistance. We develop a material design strategy that might allow the scale-up of CO_2_ electrolysis to an industrial size and enable the sustainable production of hydrocarbons.

## Introduction

The European Union has set the goal to become climate-neutral by 2050 in an attempt to limit the increase of average global temperature to 1.5 °C.^1^ To meet the demand for hydrocarbon chemicals and fuels without relying on fossil feedstocks, the industrial and transport sectors will require new production processes that can be powered by intermittent wind and solar power. One possible pathway involves capturing CO_2_ directly from the atmosphere^2^ or the ocean^3^ and converting it to useful chemical building blocks, such as C_2_H_4_, CO, or HCOOH, using electrochemical CO_2_ reduction (CO_2_R). These building blocks could then be further upgraded into plastics, fuels, or chemical intermediates using established chemical processes such as Fischer–Tropsch synthesis or methanol synthesis.^4,5^

The transfer of CO_2_R from the lab scale (cm^2^ size) to an industrial scale (m^2^ size) requires a scalable reactor design that enables high current density and high Faradaic efficiency.^4^ For illustration, reconverting 1000 Mt of CO_2_ emission of the EU transport sector in 2020^1^ with a CO_2_ electrolyzer operating at 200 mA cm^–2^ and a Faradaic efficiency of 85% would require a geometric electrode area of 30,000 km^2^—the size of Belgium. To date, the largest CO_2_ electrolyzer has an electrode area of only 100 cm^2^.^6,7^ To bridge this tremendous gap between the scale required to make an impact on climate change and the state of the art, researchers in the field of CO_2_R have adopted carbon-based gas diffusion electrodes (GDEs) from the mature field of polymer electrolyte fuel cells.^8^ The adoption of this electrode type has been an important step to intensify the process by overcoming CO_2_ mass transfer limitations in aqueous solutions. As a consequence, it is now possible to reach industrially relevant current densities (>200 mA cm^–2^) while limiting the undesired hydrogen evolution reaction (HER).

In a typical GDE, gaseous reactants diffuse through the gas diffusion layer (GDL), which consists of the carbon fiber substrate (CFS) and the microporous layer (MPL). The CFS is impregnated with PTFE to increase the hydrophobicity. Typically, the pores of the CFS have a size of 10 μm^9^ or larger and are manufactured into unique microstructural arrangements using various mechanical methods, such as weaving or hydroentanglement. The MPL, a composite layer made out of carbon black and PTFE, plays an important role in keeping the CFS dry because its small, hydrophobic pores (<0.1 μm)^10^ require high liquid overpressure to flood with liquid. This layer also provides electrical conductivity and support for the catalyst layer (CL).^8,11^ Electrochemical reactions take place in the CL, which exchanges gaseous species through the pore network of the GDL and exchanges ionic species with the adjacent liquid/ionomer phase.^12^

Generally, the research on carbon-based GDEs has been geared toward fuel cell applications, where the produced water has to be drained through the GDL to the gas channel to prevent flooding of the GDE.^13^ The flooding of the GDE, which is the saturation of the pores with liquid, is detrimental to the effective diffusivity.^14,15^ In contrast to fuel cells, CO_2_R does not produce water at the CL that has to be transported through the GDL. Therefore, a GDE design geared toward CO_2_ electrolysis should support high mass transfer between the gas channel and the cathode CL to ensure the supply of gaseous reactants (CO_2_ or H_2_O vapor) and the removal of gaseous products (CO, C_2_H_4_, or H_2_). This GDE design, in addition, should prevent the intrusion of liquids to ensure a high resistance against electrolyte flooding. This requires understanding of the design of GDEs, which involves many adjustable parameters, e.g., the microstructure of the CFS (carbon paper, carbon cloth, or nonwoven),^16,17^ the thickness, or the composition of the different layers,^18,19^ which all influence important properties like electrical conductivity,^16^ wettability,^9,20^ or diffusivity.^21^

Gas-fed CO_2_ electrolyzers with flowing catholytes have demonstrated high current densities while maintaining a high Faradaic efficiency for the CO_2_R reaction.^11,22−24^ As the GDE is in direct contact with the liquid electrolyte, the supply of water molecules for the CO_2_R reaction is no concern for this design. The flooding of the GDE with an electrolyte, however, is a major practical challenge for scale-up because the separation of the gas and liquid phases is being maintained only through the hydrophobic interfacial forces of the GDE. While it might be possible to control the differential pressure between gas and liquid to prevent flooding at a lab scale (height ≤ 10 cm),^25,26^ it becomes increasingly difficult to maintain uniform conditions over the height of the electrode at a larger scale.^27,28^ In large cells or stacks of cells, hydrostatic pressure differences are much more significant and make (local) pressure differences between gas and liquid phase inevitable. These pressure differences will lead to the flooding of the GDE in the regions of the reactor in which the capillary pressure of the pores is exceeded and consequently limit the scalability. For example, Jeanty et al. investigated the scale-up of a reactor with a flowing catholyte at a current density of 150 mA cm^–2^. The Faradaic efficiency for CO, FE_CO_, decreased from 66 to 53% after increasing the electrode area from 10 to 100 cm^2^. They attributed this decrease to the nonuniformity in reaction conditions due to GDE flooding and electrolyte breakthrough to the gas compartment.^7^

Gas-fed CO_2_ electrolyzers with membrane electrode assemblies (MEA) feature a membrane that is in direct contact with the cathode GDE. This configuration creates a physical barrier between the electrolyte and the GDE. Although this reactor concept has demonstrated high current densities with high FE_CO_,^6,29,30^ promising for scale-up and stacking,^29^ an inherent challenge of the MEA design is supplying the right amount of H_2_O to the cathode as a source of protons. For example, Berlinguette et al. showed that an insufficiently humidified CO_2_ feed can lead to rapid decay of cell performance after only 1 h of operation,^31^ while an excess of H_2_O at the cathode can also lead to performance decreases.^32^ Hence, water management remains an issue in MEA-based CO_2_ electrolyzers as well. Salt formation in gas channels is also frequently reported.^29^ This phenomenon can be mitigated by periodically flooding the gas channel with water^29,30^ and therefore still requires a detailed understanding of the flooding mechanisms of GDEs.

While most CO_2_ electrolysis research has been carried out at a scale of ≤10 cm^2^ and repurposed GDLs from fuel cell applications, only a couple of studies focused on improving the GDE structure.^11,33,34^ The scale-up of gas-fed CO_2_ electrolyzers to a scale of m^2^, however, requires the design of new materials that address the unique challenges of CO_2_R.

In this work, we investigate the effect of the GDE structure on the CO_2_R performance at commercially relevant current density in a gas-fed electrolyzer with a flowing catholyte. We investigate for the first time the effect of the GDE structure on the resistance against electrolyte flooding/breakthrough due to pressure differences between the gas and the liquid phase and how the structure impacts the formation of gaseous products in CO_2_ electrolyzers. We deposited a Ag catalyst layer on a selection of commercial GDL materials with different CFS structures (paper, nonwoven, and cloth) and thicknesses (250–450 μm). Additionally, we investigate how cracks in the MPL affect the flooding resistance and mass transfer properties of a GDE. Our analysis helps researchers select more suitable GDEs for their lab experiments using gas-fed CO_2_ electrolyzers with an MEA configuration or flowing catholyte configuration. We suggest a promising design strategy to improve carbon-based GDEs, which may be critical for the intensification and scale-up of electrochemical CO_2_ reduction.

## Experimental Methods

We prepared GDEs from a selection of commercial GDL substrates. We characterized their physical properties and tested their electrochemical performance in a gas-fed CO_2_ electrolyzer with a flowing catholyte.

### Preparation of GDE Samples

We have selected seven commercial GDL materials that varied in thickness and CFS structure (Table 1). Carbon papers are brittle materials, which are made of short carbon fiber fragments and carbonaceous binders.^35^ The TGP-H carbon papers (Toray) have similar porosity, ϵ_G,CFS_, and tortuosity, τ_G,CFS_, for their CFS. Therefore, these materials allowed us to isolate the effects of CFS thickness, δ_CFS_ (190–370 μm). In comparison, the SGL carbon papers have a larger average pore radius, *d̅*_pore_, and a wider pore size distribution (PSD). This is also reflected by their higher porosity and lower tortuosity. The LT1400W (ELAT) is a flexible carbon cloth, which has been woven from carbon fiber bundles. The woven structure results in a bimodal PSD, which has large pores (85 μm) between the fiber bundles and small pores (10 μm) between individual fibers. The H23C6 (Freudenberg) has a nonwoven CFS structure and a crack-free MPL. The carbon fibers of this GDL have been partially entangled with high pressure water jets during the production process (hydroentanglement). This procedure gives the material flexibility and a dense packing, which results in a small average pore size with a narrow PSD (16 ± 16 μm). In conclusion, the studied GDLs exhibit the following trend from wide to narrow PSD: cloth > SGL paper > Toray paper > nonwoven (Figure S1).^17,36^

**Table 1:** Commercial GDL Types with Different CFS Structures Obtained from Fuel Cell Store (USA)^g^

Material	TGP-H-060	TGP-H-090	TGP-H-120	SGL 22BB^a^	SGL 39BC^b^	LT1400W	H23C6^e^
Manufacturer	Toray	Toray	Toray	SGL	SGL	ELAT	Freudenberg
ϵ_G,CFS + MPL_^16^	-	-	-	37%	53%	63%^c^	46%
τ_G,CFS + MPL_^16^	-	-	-	2.9	1.9	-	5.0
δ_CFS + MPL_	250 μm	340 μm	430 μm	215 μm	325 μm	454 μm^c^	250 μm
Carbon fiber substrate (CFS) properties							
Structure	Paper	Paper	Paper	Paper	Paper	Cloth	Nonwoven
δ_CFS_	190 μm	280 μm	370 μm	190 μm	300 μm	406 μm^c^	210 μm^f^
*d̅*_pore_(17)	26 ± 20 μm	-	-	-	32 ± 30 μm	10, 85 μm^d^	16 ± 16 μm
ϵ_G,CFS_^16^	63%	67%	62%	66%	71%	-	-
τ_G,CFS_^16^	2.8	2.6	2.5	1.5	1.3	-	-
Microporous layer (MPL) properties							
δ_MPL_	60 μm	60 μm	60 μm	25 μm	25 μm	48 μm	40 μm

a 22BB alternative names: 25BC, 29BB; CFS data for type without MPL: 25BA.

b 39BC alternative names: 35BC, 39BB; CFS data for type without MPL: 35BA.

c FuelCellsEtc GDL comparison table.

d Bimodal pore size distribution with about 10 and 85 μm peak diameters; based on Nuvant ELAT cloth.^36^

e H23C6 alternative name: H2315 I2C6.

f CFS thickness according to supplier data sheet for type without MPL: H2315.

g The CFS of the Toray papers TGP-H-XX0 had been wet-proofed with 8–9 wt % PTFE. They were supplied to us with an MPL composed of 33–35 wt % PTFE. The CFS of the SGL papers had been wet-proofed with 5 wt % PTFE; the MPL was wet-proofed with 23 wt % PTFE. LT1400W and H23C6 had also been impregnated with PTFE, but no data were available on the exact contents. The thickness of the different layers, δ_i_, was obtained from specification sheets issued by the supplier and manufacturers. The mean pore diameter of the CFS, *d̅*_pore_, was reported by Parikh et al.^17^ The gas phase porosity, ϵ_G, i_, and tortuosity, τ_G, i_, were obtained from El-kharouf et al.^16^ Unavailable data are denoted as hyphens.

The GDEs were prepared by depositing the CL with a custom-made automated airbrush coating system (Figure S2). The target catalyst loading was 1 mg Ag cm^–2^. The solid composition was 80 wt % Ag and 20 wt % Nafion 521 ionomer. To prepare the sample, we cut the GDL to size, covered it with a 3 cm × 3 cm mask, and fixed it to the heating plate (130 °C) of the system. To prepare the catalyst ink, we added 33 mg of Ag nanopowder (20–40 nm, 99.9%, Alfa Aesar), 2.1 mL of water, 2.1 mL of isopropyl alcohol, and 180 μL of Nafion D-521 dispersion (5 wt %, Alfa Aesar) into a glass vial. We homogenized the ink for 30 min in a sonication bath. Then, we used the 2D-motorized stage to spray the ink evenly onto the MPL side of the GDL with an airbrush.

### Physical GDE Characterization

The microstructure of each GDL was visualized with scanning electron microscopy (SEM) at three different locations of the CFS and MPL.

The wettability of the different GDE layers was quantified by measuring the static contact angle. For each sample, we deposited a 10 μL water droplet at five different locations of the surface. After recording an image, we extracted the contact angle with the image processing software ImageJ.

The flooding resistance of GDL and GDE was determined by observing the gas–liquid flow regime through a transparent flow cell as a function of differential pressure, Δ*p*. We placed the sample in a flow cell (Figure S10). Then, we pumped liquid into the liquid compartment. Water was used for the GDL samples; 1 M KHCO_3_ was used for the GDE samples. While gradually increasing the liquid backpressure and keeping the gas pressure constant, we observed the gas–liquid flow regime at the sample interface at both sides. We recorded the Δ*p* between the gas and liquid compartments when a transition of the flow regime occurred (gas breakthrough, no breakthrough, or liquid breakthrough). For more details on the exact procedure for the GDL and GDE samples, see Section 6 of the SI.

The CO_2_ permeability was determined by measuring the pressure drop over the GDL as a function of the CO_2_ flow rate. We installed the GDL in a flow cell (Figure S10) and forced the gas to flow through the sample by closing the gas outlet (Figure S11). We plotted the CO_2_ flow rate against the recorded pressure drop according to Darcy’s law^37^ to determine the permeability constant, *P*_CO_2__, from the slope of the resulting linear curve.

### CO_2_ Electrolysis Procedure

**Figure 1:**
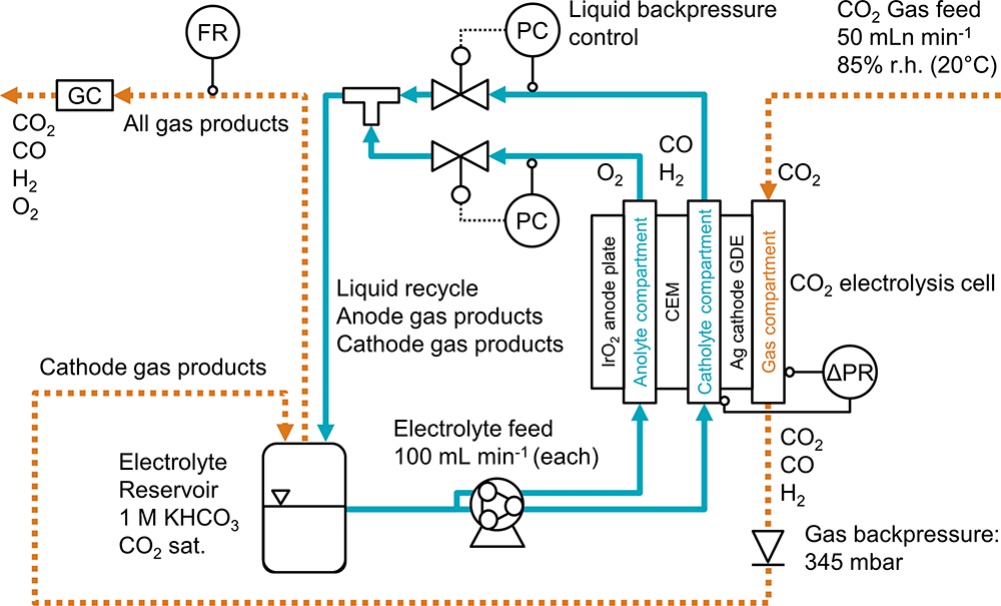
Process flow diagram for the CO_2_ electrolysis setup with differential pressure control. The anolyte and catholyte compartments were separated with a cation exchange membrane (CEM). The backpressure of both electrolyte streams was controlled (PC) before the two liquid streams were combined and recirculated. The Δ*p* across the GDE was measured between the catholyte and gas compartment (ΔPR). The Faradaic efficiency was determined by recording the flow rate (FR) with a mass flow meter (MFM) and analyzing the gas composition by gas chromatography (GC).

The CO_2_ reduction performance was measured with an automated electrolysis setup (Figure 1). We recirculated 1 M KHCO_3_ through the anolyte and catholyte compartments with a peristaltic pump. The humidified CO_2_ gas feed flowed through the gas compartment, whose backpressure was set by the cracking pressure of a check valve at the outlet. The liquid backpressure was controlled by electronic valves to obtain a flow-by regime (no breakthrough) when we applied a current density of −200 mA cm^–2^ to the cathode GDE. The product gases in the catholyte, anolyte, and gas stream were collected in the headspace of the electrolyte reservoir. We recorded the flow rate (FR) of the product gas mixture with a mass flow meter (MFM). A gas chromatography system (GC) quantified the product gas concentration from three injections. We calculated the Faradaic efficiencies for the major products CO and H_2_. The procedure is described in more detail in Section 8 of the SI. Preliminary experiments with an SGL 39BC GDE showed that the CO_2_ reduction performance remained stable for at least 2 h, which is significantly longer than the short sampling period of 10 min required to carry out three GC injections (Section 10 of the SI).

### Overall O_2_ Mass Transfer Coefficient

The limiting overall O_2_ mass transfer coefficient was measured as a proxy for the CO_2_ mass transfer coefficient. We studied the O_2_ flux induced by the oxygen reduction reaction (ORR) because it simplifies the analysis by avoiding the competing HER reaction (further discussion below). We installed the GDE in the flow cell (Figure S10) and supplied pressurized air as the gas feed (Figure S21). We carried out linear sweep voltammetry between 0 and −2 V vs SHE at a scan rate of 20 mV s^–1^. We extracted the limiting current density for the ORR from these scans and used it to calculate the corresponding limiting overall mass transfer coefficient.

## Results and Discussion

Our study revealed a number of relationships between physical properties of the GDE materials and the resulting flooding resistance and electrochemical performance.

### Microstructure and Wettability Determine Flooding Resistance

**Figure 2:**
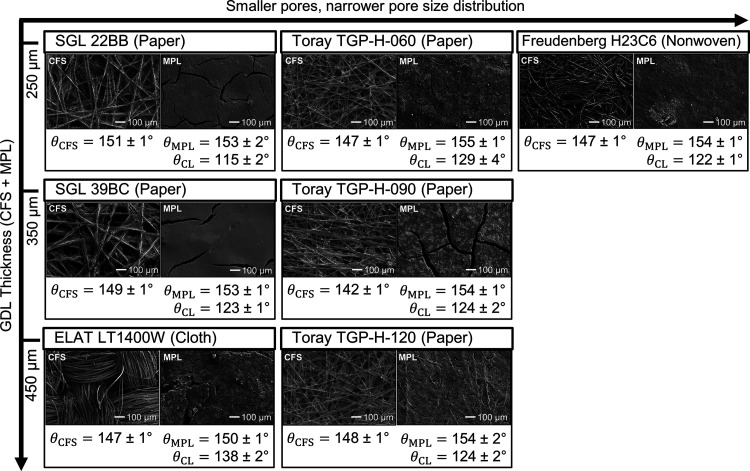
Microstructure and wettability results: SEM images of CFS and MPL at 100× magnification. Static contact angles, θ_i_, of the carbon fiber substrate (CFS), microporous layer (MPL), and catalyst layer (CL). The presented contact angles are an average of five measurements at random locations ± the standard error.

The SEM images illustrate the differences in microstructure between the materials (Figure 2). We arranged the materials according to the GDL thickness and the pore size distribution (PSD) of their CFS. The SGL carbon papers have a coarser structure than the Toray papers, which is in good agreement with the narrower PSD expected for Toray papers (Figure S1). The ELAT carbon cloth exhibits large pores between the fiber bundles. The nonwoven H23C6 has densely packed CFS with entangled fibers. Except for the H23C6, all GDLs show large cracks in the MPL with a size of tens of μm. Additional SEM images (Figure S4) let us estimate a CL thickness of 3.5 ± 0.2 μm. The primary Ag particles (79 ± 17 nm) formed larger agglomerates (200–1200 nm) embedded in a Nafion ionomer matrix (Figure S5).

The GDLs show little difference in their initial static contact angles (Figure 2). The CFS of all materials was highly hydrophobic (θ_CFS_= 142–151°), which is in good agreement with literature.^18^ The MPLs are even more hydrophobic (θ_MPL_= 150–155°) because their PTFE content is higher than that of the CFS. The higher PTFE content in the MPL of the Toray papers (33–35 wt %) than of the SGL papers (23 wt %) does not seem to increase the contact angle significantly. This is consistent with studies in the literature reporting that the effect of PTFE content on the wettability levels off after a loading of 10–20 wt % is exceeded.^38,39^ Nominally, the deposited CLs consist of 80 wt % Ag and 20 wt % Nafion. Because these components are more hydrophilic than carbon or PTFE,^40^ the surface of this layer shows a lower contact angle (θ_CL_= 115–138°). Note that the quantitative measurements of contact angles on rough surfaces are challenging (see Section 5 of the SI for a detailed discussion). For example, rough surfaces can lead to an increase of the effective contact angle according to the Cassie–Baxter model.^41,42^ This could explain why the LT1400W exhibits a higher θ_CL_ than the other materials.

We observed three different regimes of the two-phase flow at the GDE. These three flow regimes depend on the differential pressure between the liquid and the gas compartment, Δ*p* = *p*_L_ – *p*_G_: (i) Gas breakthrough occurs when Δ*p* is below the threshold for gas breakthrough, Δ*p*_G_^*^ (flow-through). (ii) No breakthrough occurs when Δ*p* is increased and the fluid phases are separated (flow-by).^25^ (iii) Liquid breakthrough occurs when Δ*p* exceeds the liquid breakthrough pressure, Δ*p*_L_^*^, which is also called the percolation threshold.^43^ Based on these flow regimes, we define the flow-by pressure window, Δp* = Δ*p*_L_^*^ – Δ*p*_G_^*^, as a metric for flooding resistance.

**Figure 3:**
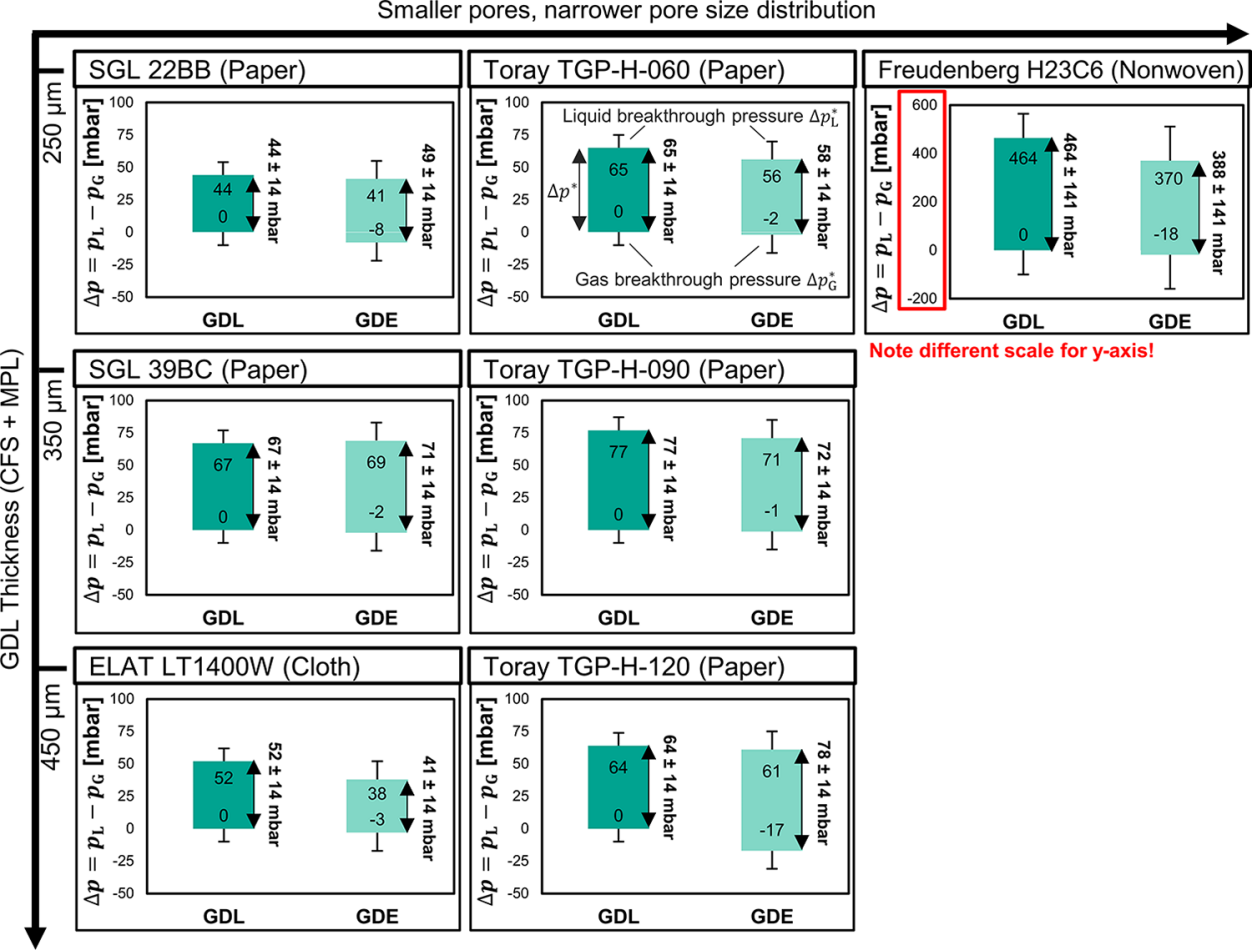
Flooding resistance results: determination of flow-by pressure window, Δp* = Δ*p*_L_^*^ – Δ*p*_G_^*^, for uncoated GDL (MPL + CFS) and coated GDE (CL + MPL + CFS). Upper limit of bar chart: liquid breakthrough pressure, Δ*p*_L_^*^. Lower limit: gas breakthrough pressure, Δ*p*_G_^*^. The gas breakthrough pressure limit of the uncoated GDL samples was not measured; we assume that it was 0 mbar. The arrows next to the bar charts indicate the corresponding flow-by pressure window, Δp*. The listed values are based on measurements of a single sample. For the breakthrough pressures, we estimated errors of σ_*p*_G_^*^_= 10 ± mbar and σ_*p*_L_^*^_= 10 ± mbar of all GDEs based on the work of Mortazavi et al. (except H23C6)*.*^19^ For H23C6, we estimated errors of σ_*p*_G_^*^_= 100 ± mbar and σ_*p*_L_^*^_=100 ± mbar based on the work of Leonard et al.^44^ The error of the flow-by pressure window, Δp*, was estimated with the Gaussian error propagation .

The flow-by pressure window, Δp*, of most commercial GDL materials falls within a range of 40–80 mbar (Figure 3). This relatively low value implies that the scale-up of a flow-by electrolyzer would be limited to a height of about 41–81 cm. In practice, the height would have to be even smaller to make the process robust against variations in the material properties (σ_Δp*_= ± 14 mbar) and the limited accuracy of pressure control at the process level.

**Figure 4:**
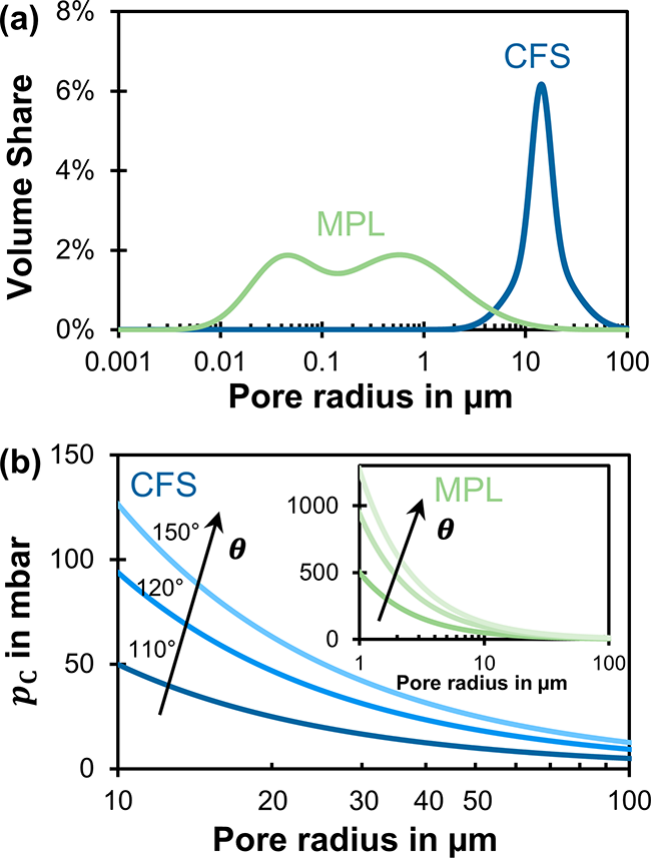
(a) Typical pore size distribution of a SGL carbon paper with MPL. The *y* axis shows the share of the total pore volume for a pore with radius *r*.^10^ (b) Capillary pressure, *p*_C_, calculated with the Young–Laplace equation *p*_C_ = – 2γ cos θ *r*^–1^, in which *r* is the cylindrical pore with radius, θ is the wall contact angle (110°, 130°, 150°), and γ is the electrolyte surface tension (H_2_O at 20 °C: γ = 73 mN m^–1^). The pore floods with liquid when the differential pressure acting on the pore exceeds the capillary pressure: Δ*p* = *p*_L_ – *p*_G_ ≥ *p*_C_.

The application of the CL shifts the pressure window, Δp*, to more negative values (Figure 3) without affecting the width significantly. This negative shift can be seen in the lower Δ*p*_L_^*^, as the comparison between the upper limit of the pressure window of the GDL samples with the upper limit of the GDE samples shows. This phenomenon can be explained by the decrease of θ on the liquid side (Figure 2). According to the Young–Laplace equation (Figure 4b), the higher hydrophilicity lowers the capillary pressure, *p*_C_, which eases the flooding of pores in the GDE.

The flow-by pressure window, Δp*, is an order of magnitude smaller for materials with cracks in the MPL (Figure 3). If no cracks are present (H23C6), the intruding liquid has to pass through the pores of the MPL. The pores of the MPL require a larger liquid pressure to be flooded because they are a lot smaller than the pores of the CFS (Figure 4). However, the largest pores determine the liquid breakthrough pressure, and cracks count as extremely large pores in the MPL. If cracks are present (all other GDEs), the MPL is bypassed and the liquid breakthrough pressure is determined by the pores of the CFS.

**Figure 5:**
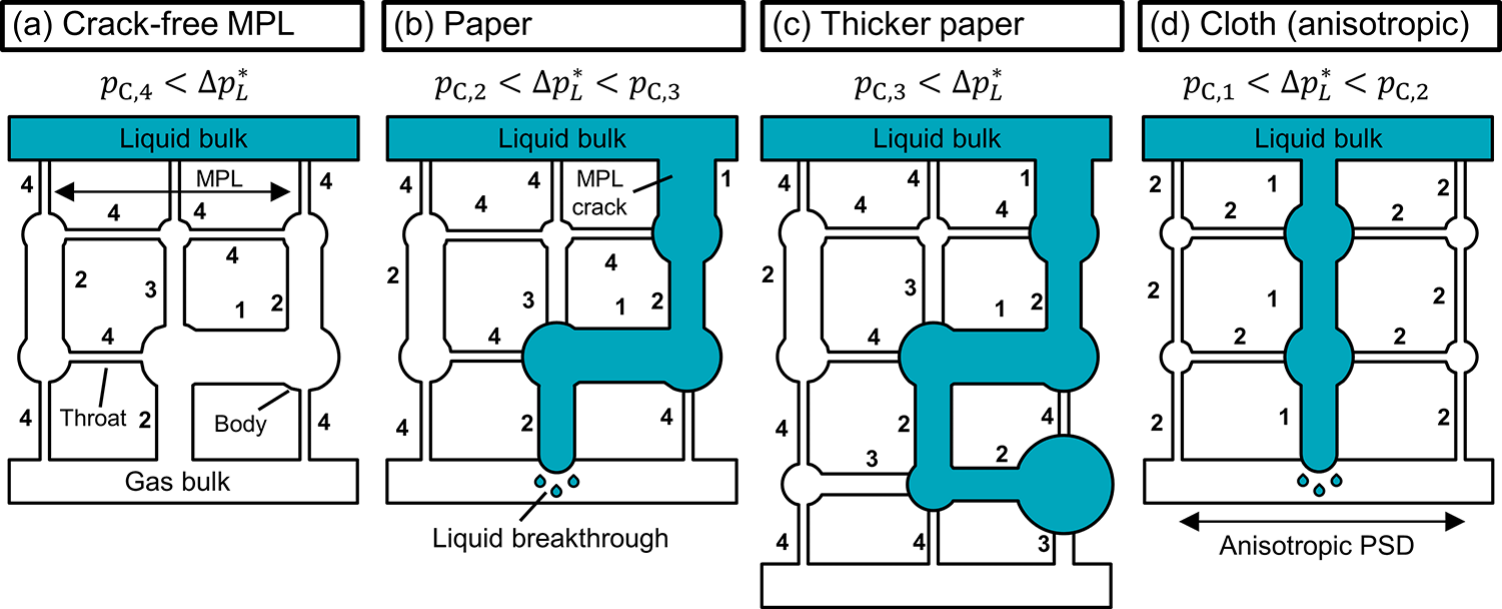
Schematic pore network models^45−47^ representing different GDL materials. The spatial connectivity of the pores determines the percolation flow path and the liquid breakthrough pressure, Δ*p*_L_^*^. The relative order of capillary pressures is *p*_C,1_ < *p*_C,2_ < *p*_C,3_ < *p*_C,4_. (a) Crack-free MPL: The network remains dry until the liquid exceeds the high capillary pressure of the MPL: *p*_C,4_. (b) Carbon paper: The highest capillary pressure in the flow path, *p*_C,2_, determines Δ*p*_L_^*^. (c) Carbon paper with increased thickness: Compared with (b), the longer percolation pathway increases the probability of encountering pores with higher *p*_C,3_. (d) Carbon cloth: The wide pore size distribution with anisotropic structure leads to a preferential breakthrough path along pores with low capillary pressure *p*_C,1_. Adjacent pores with higher *p*_C,2_ remain dry and allow gas diffusion.

The two different percolation flow paths, with and without cracks in the MPL, are illustrated in Figure 5a,b using schematic pore network models.^19,45,46^ Each network consists of pore bodies (circles) and throats (rectangles). The throats restrict fluid intrusion according to their capillary pressure, *p*_C,i_. The spatial connectivity of the pores determines the percolation flow path and the liquid breakthrough pressure, Δ*p*_L_^*^. For the material with the crack-free MPL (Figure 5a), the narrow pores of the MPL prevent liquid intrusion into the gas-filled network until the high capillary pressure of *p*_C,4_ is exceeded. For the material with cracks bypassing the MPL (Figure 5b), Δ*p*_L_^*^ drops to *p*_C,2_, which is the highest capillary pressure in the flow path of the percolating liquid.

The flow-by pressure window, Δp*, of the GDE is also increased by a thicker CFS. This is illustrated by the data of the SGL and Toray papers (Figure 3). For example, the Δp* improves from 58 mbar for the thinnest Toray paper (TGP-H-060) to 78 mbar for the thickest (TGP-H-120). This trend is in good agreement with the liquid breakthrough pressures recorded by Mortazavi and Tajiri, who explain that a thicker GDL has a higher probability to have small, hydrophobic pores in the percolation flow path.^19^ We illustrate this phenomenon with the pore network model in Figure 5b,c: The additional layer in the pore network of the thicker paper (Figure 5c) increases the probability that the liquid is stopped by a pore with *p*_C,3_, which increases the liquid breakthrough pressure, Δ*p*_L_^*^, without affecting the gas breakthrough pressure, Δ*p*_G_^*^.

GDLs with broader pore size distributions exhibit a lower flooding resistance (Figure 3). This effect is most apparent for the LT1400W carbon cloth, which has a similar thickness to the TGP-H-120 carbon paper but has a much smaller Δp*: 41 mbar vs 78 mbar. The effect of larger CFS pores is enhanced by the anisotropic PSD of the cloth: The large pores are located in between the fiber bundles and go all the way through the cloth, while the small pores are located inside of the fiber bundles. The large pores, therefore, offer a preferential percolation flow path, which bypasses smaller pores with higher capillary pressure (Figure 5d). The effect of wider pore size distributions becomes clear—although to a lesser extent—by comparing the carbon papers SGL 22BB and TGP-H-060. Here, the SGL 22BB has a wider pore size distribution, which results in a lower Δp* of 49 mbar than 58 mbar. We note that the Toray papers had a thicker MPL than the other GDL materials, which could convolute the effects of a narrower PSD and of a thicker MPL on the flooding resistance. We argue, however, that the properties of the CFS are more significant because the MPL offers little flooding resistance due to its large cracks. In summary, broader PSDs lead to a lower flooding resistance; however, they can also be advantageous because a large fraction of pores remains accessible for gas diffusion even if liquid breakthrough is occurring.^38^

### Microstructure Determines Mass Transfer and CO_2_ Reduction Performance

As a mass transfer metric, the limiting current density for the CO_2_ reduction is a valuable metric. However, the H23C6 was not stable during CO_2_ electrolysis at a current density of −200 mA cm^–2^ (discussion further below). To isolate the mass transfer of the gaseous species from other factors (such as GDE stability), we measured the limiting overall O_2_ mass transfer coefficient, *k*_O_2__, in flow-by mode as a proxy for the CO_2_ mass transfer. The CO_2_R and the oxygen reduction reaction (ORR) are both subject to mass transfer limitations at sufficiently high current densities. The derived mass transfer metrics, however, can only be compared qualitatively between GDL substrates because the solubility and diffusivity of the two gases differ.

**Figure 6:**
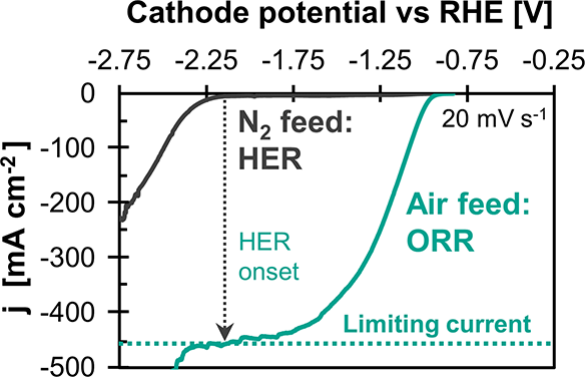
Limiting O_2_ mass transfer as proxy for CO_2_ mass transfer: Example LSV scan for SGL 39BC loaded with 1 mg Ag cm^–2^. HER, hydrogen evolution reaction; ORR, oxygen reduction reaction; O_2_ + 2 H_2_O + 4 e^–^ → 4 OH^–^. The cathode potentials were corrected for the *iR*-drop between the reference electrode and the cathode. To reach sufficiently high currents with our potentiostat, we used a 6 M KOH electrolyte due its high conductivity.

**Figure 7:**
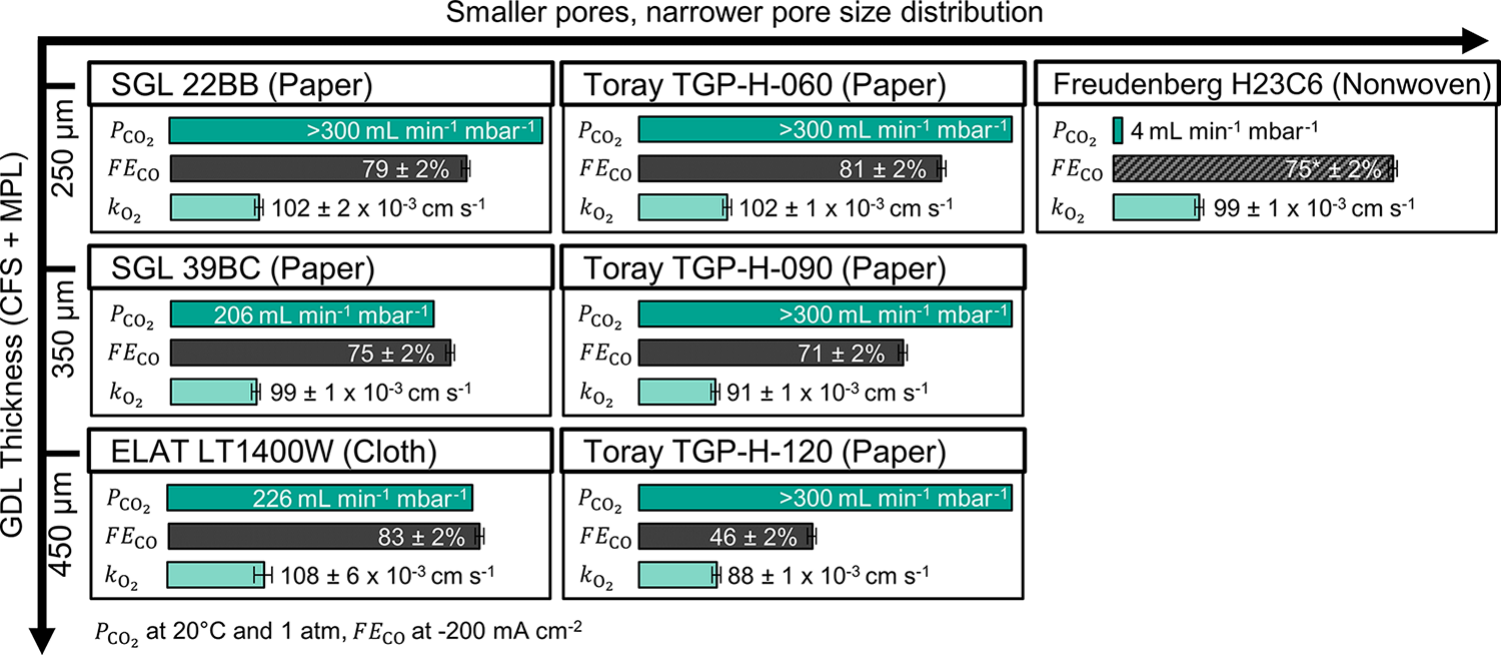
Mass transfer characterization results. Uncoated GDL samples (CFS + MPL): The CO_2_ permeability constant, *P*_CO_2__, of the Toray papers and SGL 22BB were out of range of our experimental setup (300 mL min^–1^ mbar^–1^). GDE samples (flow-by mode): FE_CO_ at 200 mA cm^–2^, *k*_O_2__ is the limiting overall O_2_ mass transfer coefficient (proxy for CO_2_ mass transfer). Note that Freudenberg H23C6 is unstable at 200 mA cm^–2^. The FE_CO_= 75 ± 2% is a hypothetical value based on the data for SGL 39BC because the *k*_O_2__ of the two materials is equivalent.

We determined *k*_O_2__ from the limiting current density of the oxygen reduction reaction (ORR) extracted from an LSV scan (Figure 6). This reaction is commonly performed with Ag-based GDEs for chlor-alkali electrolysis with oxygen-depolarized cathodes.^48^ The onset potential of the ORR is much higher (less negative) than for the competing HER, as the comparison of the LSV scan for an air feed (21 vol % O_2_) with a N_2_ feed illustrates. This leads to a distinct current density plateau at which the oxygen transfer to the CL determines the reaction rate. We used this limiting current density, therefore, to calculate the corresponding overall mass transfer coefficient, *k*_O_2__. This metric describes the limiting transport of O_2_ from the gas bulk, through the different GDE layers, to the surface of the catalyst (details of the data processing are explained in Section 9 of the SI). The resulting values for *k*_O_2__ are presented together with the other mass transfer and electrolysis metrics in Figure 7.

Our mass transfer and electrolysis results suggest that convective mass transfer (permeation) might be of secondary importance for our electrolysis conditions (Figure 7). The CO_2_ permeability constant, *P*_CO_2__, showed a poor correlation with FE_CO_ or the limiting overall O_2_ mass transfer coefficient, *k*_O_2__. For example, we measured a lower *P*_CO_2__ for SGL 39BC in comparison with TGP-H-120, but the SGL 39BC exhibits a higher FE_CO_ and *k*_O_2__. While a more quantitative analysis is precluded by the limited range of our *P*_CO_2__ data, it seems plausible, however, that the mass transfer occurs primarily by gas diffusion through the CFS and by a combination of gas and Knudsen diffusion through the MPL, as is the case in hydrogen fuel cells.^49^

An increase in GDL thickness limits the mass transfer significantly. This can be clearly seen by the trends of FE_CO_ (recorded at 200 mA cm^–2^) for the SGL and Toray carbon papers (Figure 7). When comparing the thin TGP-H-060 with the thicker TGP-H-120, for example, the FE_CO_ drops from 81 to 46%. Kenis et al. reported a similar trend in their study on the GDE structure.^11^ We observe this decrease in FE_CO_ because the supply of CO_2_ to the CL is restricted by the thicker CFS. The CO_2_ diffusion rate, therefore, is unable to keep up with the electrical current, which leads to excess electrons being consumed by the competing HER.

In contrast, CFS structures with a broader PSD allow higher mass transfer rates. This is well illustrated by the data for LT1400W and TGP-H-120 (Figure 7). Both had a similar thickness, but the carbon cloth allowed a much better FE_CO_. Another example that shows the effect of a broader PSD is the comparison of SGL 39BC vs TGP-H-090. Again, the samples have a similar thickness, but the SGL 39BC showed a better FE_CO_ of 75% in comparison with 71% recorded for the TGP-H-090. The materials with broader PSD tend to have a higher porosity, ϵ_G, CFS_, and lower tortuosity, τ_G, CFS_ (Table 1). These properties improve the mass transfer coefficient through the CFS, *k*_CO_2_, CFS_, by increasing the effective diffusivity, *D*_eff, CO_2__, according to eq 1.^50,51^
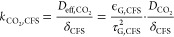
1

Our results also indicate that a large resistance to mass transfer must lie in the CL. We come to this conclusion from decomposing the overall O_2_ mass transfer coefficient, *k*_O_2__. This empirical metric is an overall mass transfer coefficient that incorporates the serial resistance over the different domains of the GDE (CFS, MPL, and CL). Figure 7 already reveals that *k*_O_2__ is not inversely proportional to the CFS thickness. If the mass transfer through the CFS were limiting, we would expect a relative mass transfer coefficient of about +100% for TGP-H-060 compared to TGP-H-120 as its porosity and tortuosity are similar (Table 1). The empirically determined *k*_O_2__, however, shows only an increase of +16% (0.102 cm s^–1^ vs 0.088 cm s^–1^). This means that an additional resistance to mass transfer must be responsible for the smaller difference. We decomposed *k*_O_2__ for SGL 22BB in Table S7 by using characterization data from fuel cell research. Based on the data of Reshetenko and Ben,^52^ we estimated the mass transfer coefficients of *k*_O_2_, CFS_= 2.60 cm s^–1^, *k*_O_2_, MPL_= 5.97 cm s^–1^, and *k*_O_2_, CL_= 0.108 cm s^–1^. We note that *k*_O_2_, MPL_ has a higher value than *k*_O_2_, CFS_ because the MPL is an order of magnitude thinner than the CFS. The much lower value for *k*_O_2_, CL_ corresponds to the CL being responsible for 94% of the mass transfer resistance of this material (Table S9). The resistance of the CL is probably so high because it is flooded with the electrolyte. The improvement of the CL resistance would, therefore, be an important topic for future research. We note that the mass transfer through the CL is probably lower for the ORR experiments than for the CO_2_R experiments. Due to the higher viscosity of 6 M KOH, the diffusivity of O_2_ in this electrolyte (0.7 × 10^–5^ cm^2^ s^–1^)^53^ is 56% lower than that of CO_2_ in 1 M KHCO_3_ (1.6 × 10^–5^ cm^2^ s^–1^)^54^ at 25 °C. In addition, the solubility of O_2_ in 6 M KOH (0.01 M)^53^ is three times lower than that of CO_2_ in 1 M KHCO_3_ (0.034 M).^54^ Quantitative predictions, however, are difficult to make because the material values in the porous Nafion matrix of the CL are likely to differ from the corresponding values for bulk electrolytes.

The nonwoven H23C6 was unstable during CO_2_ electrolysis at 200 mA cm^–2^. At these conditions, the GDE lost its hydrophobicity and the gas compartment started to flood so that we were unable to measure a representative FE_CO_ (Figure 7). Similar behavior for this GDL material has been also reported in the literature.^44,55^ Leonard et al. and Yang et al., for example, reported a degradation of carbon when the cathode potential was more negative than −0.65 V vs RHE.^44,55^ We confirmed the hypothesis that the CFS degraded experimentally. After applying a current density of −100 mA cm^–2^ at −1.2 V vs RHE for 111 min, the θ_CFS_ dropped to 131 ± 2° from its initial value of 147 ± 1°. We hypothesize that the poor stability of the Freudenberg H23C6 might be attributed to a larger number of oxygen groups at the surface of its carbon fibers relative to the other substrates (Freudenberg: 10 at % vs SGL: <1 at %).^56,57^ These oxygen functionalities might facilitate the degradation of the surface by serving as active sites for the carbon surface oxidation.^58^ The higher oxygen content probably originates from a lower degree of carbonization,^59,60^ which probably also gives this material its high flexibility. A systematic study of the degradation mechanism of H23C6 would be an important contribution for future research.

The transport through MPL cracks seems to play a secondary role for the mass transport and Faradaic efficiency during electrolysis (Figure 7). If we compare the data for H23C6 and SGL 39BC, we find that the O_2_ mass transfer coefficient of both samples was equivalent (*k*_O_2__= 0.99 cm s^–1^), although the *P*_CO_2__ was two orders of magnitude lower for the H23C6 due to the lack of cracks in the MPL. This result shows that the cracks in the MPL do not have a significant impact on the mass transfer during electrolysis. It is likely that the cracks are filled with the electrolyte during electrolysis and the transport of CO_2_ to the CL occurs through the gas-filled pore network of the MPL. Based on the equivalent values of *k*_O_2__ for these samples, we can also hypothesize that H23C6 would allow a FE_CO_= 75% at 200 mA cm^–2^ if it were stable.

### Trade-off between Flooding Resistance and Mass Transfer Limits Scalability

**Figure 8:**
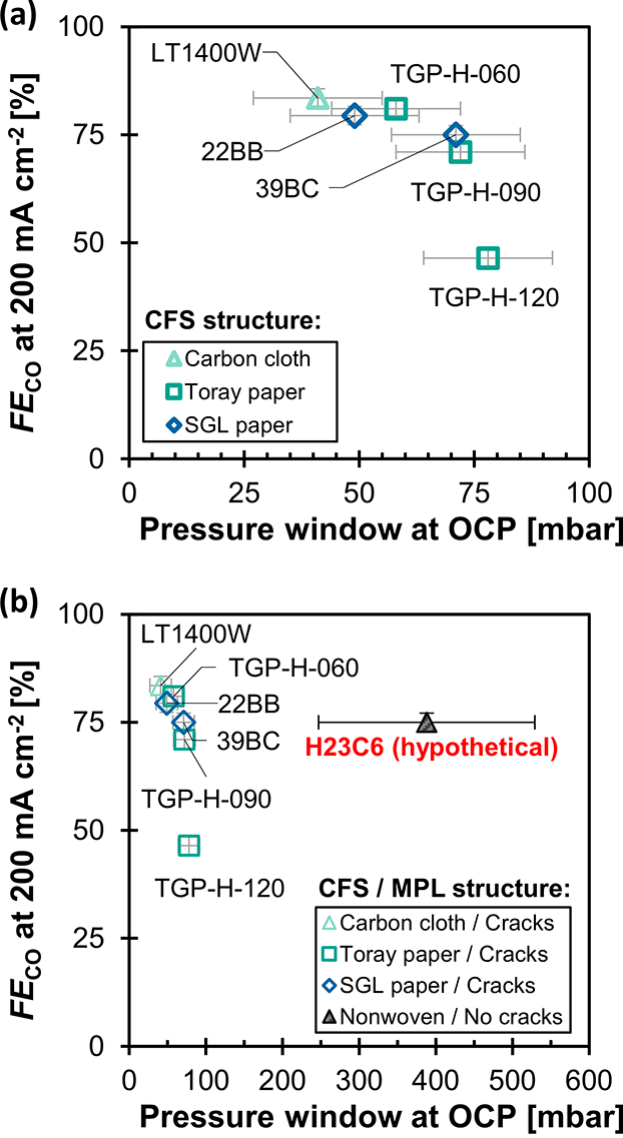
Trade-off between flooding resistance and CO_2_ mass transfer: FE_CO_ at −200 mA cm^–2^ (flow-by mode) against the flow-by pressure window, Δp*, recorded at open circuit potential (OCP). The shape and color of the markers indicate the CFS structure of each GDE. (a) Detailed view of materials with MPL cracks. (b) Comparison of materials with and without MPL cracks (H23C6). The H23C6 marker represents a hypothetical value for FE_CO_ because this GDE type experienced a complete flooding of the gas channel at −200 mA cm^–2^.

There seems to be an inevitable trade-off between the flooding resistance of the CFS at open circuit potential (OCP) and the mass transfer capabilities (Figure 8a). GDEs with a broad PSD and/or with a thin CFS achieve the highest FE_CO_ at 200 mA cm^–2^. This presents a dilemma for building larger scale reactors because these same materials exhibited the lowest pressure window (Δp*< 50 mbar). For illustration, 50 mbar of hydrostatic pressure difference corresponds to 51 cm cell height with an aqueous electrolyte in vertical orientation. Commercial alkaline electrolysis for H_2_ production operates at a similar current density (200–400 mA cm^–2^) but commonly uses plate diameters of 100–200 cm, which implies a 4–32× larger production rate per cell compared to a 51 cm tall CO_2_ electrolyzer operating at 200 mA cm^–2^.^61^ The smaller cell height of the CO_2_ electrolyzer would, therefore, imply higher capital expenditures. Using a GDE with a thicker CFS and a narrower PSD would sacrifice in terms of mass transport rate. TGH-H-120, for example, exhibits a small gain in pressure stability (Δp*= 78 mbar), but in exchange, its FE_CO_ falls below 50%.

It might be possible to avoid this trade-off between flooding resistance and mass transfer capabilities if the MPL is crack-free (Figure 8b). As already established in the previous section, the physical structure of the crack-free H23C6 allows mass transfer rates that should be able to provide a FE_CO_ of 75% at 200 mA cm^–2^ if it were electrochemically stable. This is remarkable because at the same time this material can also withstand gas–liquid differential pressures at OCP that would allow electrolysis cells with a height of more than 1 m. We hypothesize that the pressure window of the other GDEs could be greatly improved by curing the MPL cracks with a targeted application of a carbon black and PTFE mixture. Note that although the crack-free H23C6 shows that large Δp* are possible at OCP, the hydrophobicity (and thus, Δp*) decreases when a potential is applied during operation conditions due to electrowetting.^26^ The effect of electrowetting on the flooding resistance and mass transfer is a topic for future work and could shine light on the potential for other crack-free MPLs.

## Conclusions

We have studied seven commercial GDLs with a range of structural parameters (CFS structure, CFS thickness, and cracks in the MPL). The flooding behavior and mass transfer characteristics gave insight into the selection of suitable GDEs for CO_2_ electrolyzers.

The carbon cloth (ELAT LT1400W) showed the highest mass transfer for gas–liquid CO_2_ electrolysis operation because the woven fiber bundles lead to an anisotropic PSD that has a broad (bimodal) distribution in the plane of the cloth, which allows high diffusivity. Carbon papers with thinner CFS (SGL 22BB, TGP-H-060) offer slightly lower mass transfer rates due to their narrower, more isotropic pore structure. Cloths and thin papers minimize the diffusional pathway at the cost of low resistance against flooding through liquid–gas overpressure (<50 mbar).

If the CO_2_ electrolyzer with a flowing catholyte should be operated in flow-by mode, this low resistance against flooding in commercial GDLs poses serious limits on the scalability. All materials with acceptable Faradaic efficiencies for CO_2_R (>50%) at 200 mA cm^–2^ suffer from a poor flooding resistance due to cracks in the MPL. Because of hydrostatic pressure differences between the gas and liquid compartments, this poor flooding resistance would limit the maximum cell height to less than 51 cm if the electrolyzer should be operated in flow-by mode.

The only material with a crack-free MPL (H23C6) showed a very promising initial flooding resistance (>200 cm) but degraded during CO_2_ electrolysis. This degradation requires more comprehensive investigation because it remains unclear why the carbon-based GDEs differed in electrochemical stability. By using O_2_ mass transfer as a proxy for CO_2_ mass transfer, we were able to show that cracks in the MPL are not essential for high diffusion rates. The most significant resistance to mass transfer, however, was posed by the CL, which was probably flooded. Future research could optimize the performance by investigating the resistance in the CL in more detail.

The trade-off between flooding resistance and mass transfer capability has to be overcome before CO_2_ electrolyzers can be constructed at an industrial scale. Our study implies that the layers of the ideal GDE have to be optimized for different objectives: The CFS should be thin and feature a broad PSD to minimize the diffusional pathway. The MPL should be crack-free to protect the GDE from electrolyte flooding. Such a CO_2_ electrolysis-geared GDE design might enable a GDE height larger than 100 cm for gas-fed electrolyzers with flowing catholytes. An alternative pathway to industrial CO_2_ electrolysis is offered by MEA-based systems, which should be less complex to scale-up because their membrane constitutes a physical barrier against electrolyte flooding.

## Supporting Information Available

Qualitative pore size distributions; detailed experimental procedures; additional catalyst layer characterization data; technical documentation of the electrolysis setup; data processing for the O_2_ mass transfer coefficient; calculations for decomposition of the O_2_ mass transfer coefficient; description and results of the stability test (PDF)
Numerical values for all results and figures; all recorded process parameters for CO_2_ electrolysis experiments (XLSX)
