# Supplementary material for: Narrow Pressure Stability Window of Gas Diffusion Electrodes Limits the Scale-Up of CO2 Electrolyzers
Source: ACS Sustain Chem Eng. 2022 Mar 29;10(14):4683–93. doi: 10.1021/acssuschemeng.2c00195 (PMC9006256; doi:10.1021/acssuschemeng.2c00195)
Supplement: Supplementary file 1 — sc2c00195_si_001.pdf [file sc2c00195_si_001.pdf]

SUPPORTING INFORMATION

Narrow Pressure Stability Window of Gas Diffusion Electrodes limits the Scale-up of CO_2 Electrolyzers

Lorenz M. Baumgartner,[a] Christel I. Koopman,[a] Antoni Forner-Cuenca,[b] David A. Vermaas*,[a]

[a]*Department of Chemical Engineering, Delft University of Technology, Van der Maasweg 9, 2629 HZ Delft, Netherlands*

[b]*Department of Chemical Engineering and Chemistry, Eindhoven University of Technology, Het Kranenveld 14, 5612 AZ Eindhoven, Netherlands*

Email: D.A.Vermaas@tudelft.nl

Supporting Information Content:

- 29 Pages
- 10 Tables
- 25 Figures

Contents

1. General information 3

2. GDL substrates: Qualitative comparison of pore size distributions 3

3. GDE preparation 4

4. Microstructure inspection with Scanning Electron Microscopy (SEM) 5

5. Static contact angle as metric for wettability 7

6. Flooding resistance 10

7. CO_2 Permeability for uncoated GDL (CFS + MPL) 13

8. CO_2 electrolysis procedure 14

8.1 Assembly of 3-compartment CO_2 electrolysis cell 14

8.2 Operation of the CO_2 electrolysis setup 17

9. Overall O_2 mass transfer coefficient $kO2$ of GDE 22

10. Stability test for CO_2 electrolysis 27

References 28

1. General information

De-ionized water was used for all experiments. Detailed experimental results are available in the **Excel file** *SI_Baumgartner_Vermaas_2022a.xlsx* of the supporting information.

2. GDL substrates: Qualitative comparison of pore size distributions

The studied GDLs exhibit the following trends from narrow to wide pore size distributions (PSD): Nonwoven < Toray paper < SGL paper, Cloth (**Figure S1**). Note that **Figure S1** only shows the qualitative difference of the CFS types because we used different materials in this study. Forner-Cuenca *et al.* used materials without MPL and without PTFE wet-proofing. The Nuvant carbon cloth is of a different type than our LT1400W cloth.[1]

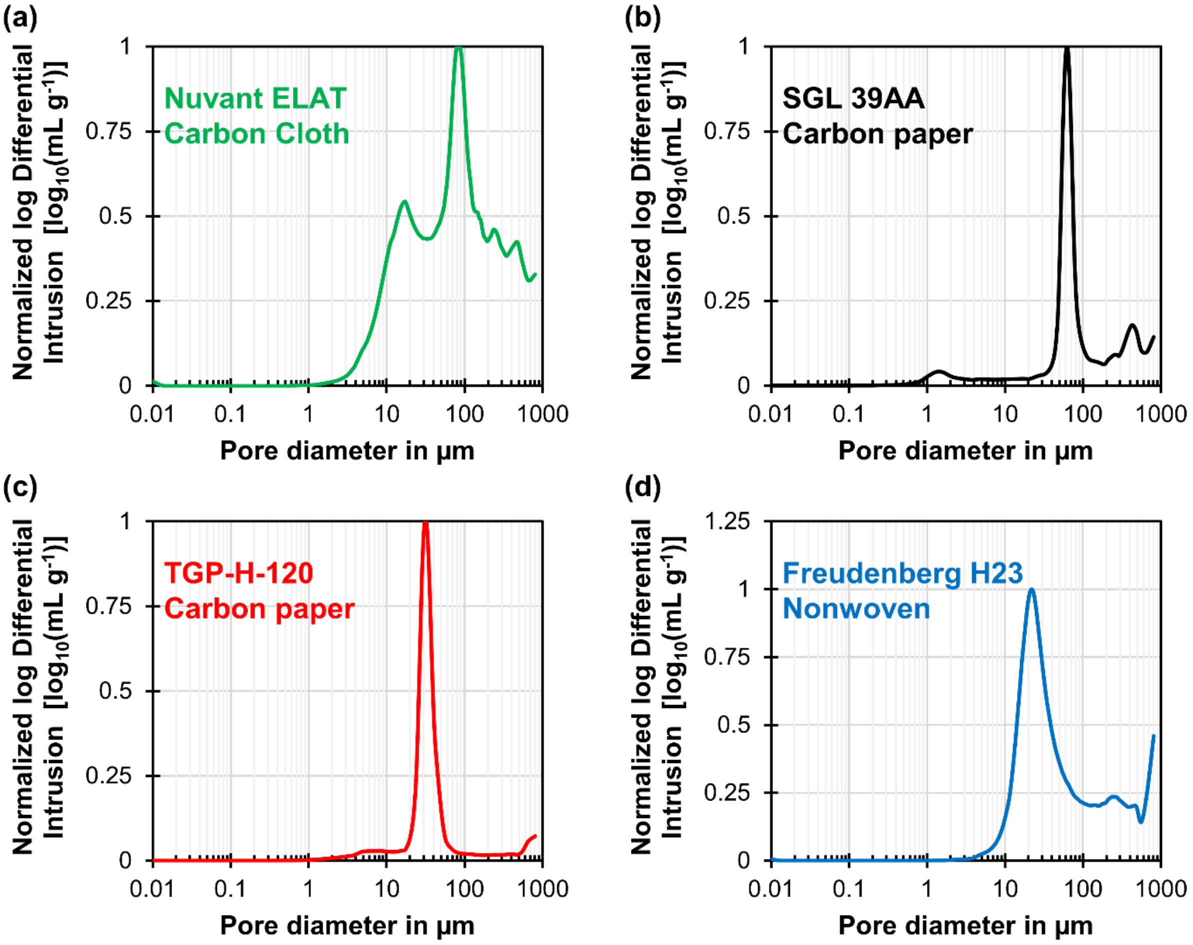

Figure S1: Qualitative comparison of the pore size distributions for the different carbon fiber substrate types. **(a)** ELAT carbon cloth **(b)** SGL carbon paper **(c)** Toray paper **(d)** Freudenberg Nonwoven. Note that the substrates shown here differ from our study because they were not impregnated with PTFE and do not have a MPL. The data is based on mercury intrusion porosimetry measurements from Forner-Cuenca *et al.*.[1]

3. GDE preparation

We prepared each GDE by depositing the CL with a tailor-made automated airbrush coating system (**Figure S2**).

Sample preparation

We cut the GDL to size a size of 3.5 cm x 3 cm, dried it for 10 min at 120°C, and weighed it in an airtight container (Kartell 034600 Polypropylene Weighing Bottles – 50 mL, Fisher Scientific). We then covered the sample with a 3 cm x 3 cm PTFE mask and fixed it to the heating plate (130°C).

Ink preparation

An example for the ink specifications is given in **Table S1**. The target catalyst loading was 1 mg Ag cm^{-2}. We selected this common catalyst loading to simplify the comparison with other studies.[2] The solid composition was 80 wt% Ag and 20 wt% Nafion 521 ionomer. The Nafion binder content of 20 wt% was selected to match the optimized content determined by Duarte *et al.*.[3] To prepare the catalyst ink, we added 33 mg of Ag nanopowder (Aerodynamic particle size (APS): 20–40 nm, 99.9%, Alfa Aesar), 2.1 mL of water, and 2.1 mL of isopropyl alcohol into a 10 mL glass vial. Then, we added 180 µL of Nafion D-521 dispersion (5 wt%, Alfa Aesar) to achieve an ink solid (Ag +Nafion) concentration of 1% g mL^{-1}. Note that we used an excess of ink to compensate for the loss of 30% ink during the deposition process. We homogenized the ink for 30 min in a sonication bath (USC500TH, VWR).

Deposition process

The ink was evenly sprayed onto the MPL side of the GDL sample with a Paasche Airbrush Set TG3 (Airbrush Services Almere, Netherlands) mounted on a custom made 2D-motorized stage (**Figure S2**).

Determination of catalyst loading

After the deposition process, we dried and weighed the coated sample for 10 min at 120°C to determine the mass of the CL. An overview of the catalyst loading per sample is given in **Table S2**.

Table S1: Example for catalyst ink specifications.

Parameter	Unit	Value
Ag nanoparticle target loading	mg cm^{-2}	1.1
Expected deposition efficiency	%	30%
Spray-coated area	cm^{2}	9
Required catalyst mass	mg	33
Weighed catalyst mass	mg	33.4
Nafion content in catalyst layer	wt%	20
Add Nafion solution (5 wt%)	mL	0.180
Ink solid concentration	w/v in g mL^{-1}	1%
Add H_2O:IPA (1:1) mixture	mL	4.175

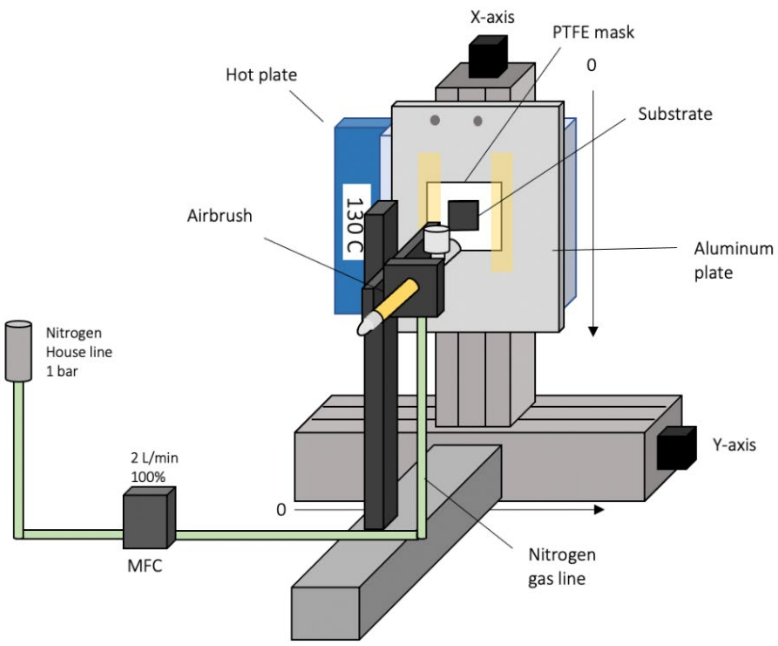

Figure S2: Catalyst layer deposition: Schematic of airbrush coating system.

Table S2: Summary of Ag catalyst loading in mg cm^{-2} for samples used in experiments to determine the Faradaic efficiency for CO, FE_{CO}, and the overall O_2 mass transfer coefficient, k_{O_2}.

Experiment	TGP-H-060	TGP-H-090	TGP-H-120	SGL 22BB	SGL 39BC	LT1400W	H23C6
FE_{CO}	1.16	1.08	1.05	1.29	1.40	1.20	1.18
k_{O_2}	1.12	1.07	1.08	1.08	1.16	1.09	1.03

4. Microstructure inspection with Scanning Electron Microscopy (SEM)

The GDE microstructure was visualized with a JSM-6010LA SEM (JEOL, Japan). The instrument was equipped with a secondary electron imaging (SEI) for morphology and a backscattered electron (BEC) detector for elemental contrast imaging.

Catalyst layer analysis

We cut a coated Freudenberg H23C6 sample with a surgical blade and looked at the edge with SEM (**Figure S3**). The SEI detector allowed to image the morphology at high resolutions. The BEC allowed us to identify the CL on top of the other layers. The brighter areas (**Figure S2 c**) indicate the fragments of the CL on the edge of the GDE. Because the CL consists of heavier Ag atoms and Nafion (sulfonated fluoropolymer), it appears brighter than the MPL and CFS. These two layers are less dense because they are composed of mostly carbon and < 20 wt% PTFE (fluoropolymer).

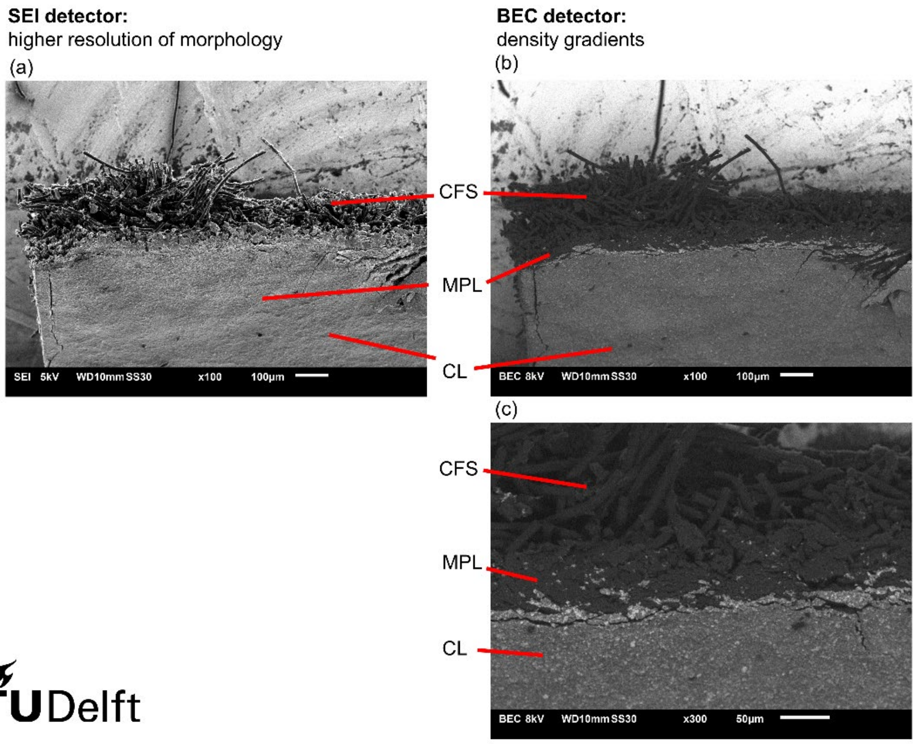

Figure S3: Exposed cross-section of GDE shows different layers: Carbon fiber substrate (CFS), microporous layer (MPL), catalyst layer (CL). **(a)** The secondary electron detector (SEI) shows the morphology at high resolution. **(b)** The backscattered electron detector (BEC) reveals density gradients, which allow the identifications of regions with differing elemental composition. **(c)** The density differences clearly distinguish the CL from the other layers.

Catalyst layer thickness

We determined the catalyst layer thickness by looking for a fragment of the CL lying on its side (**Figure S4**). The images recorded with SEI make it difficult to distinguish which particles belong to the CL (bottom row). The BEC detector let us locate a suitable fragment of the CL (top row). We measured the thickness of this particle with ImageJ at 5 random locations. The average thickness is 3.5 ± 0.2 µm.

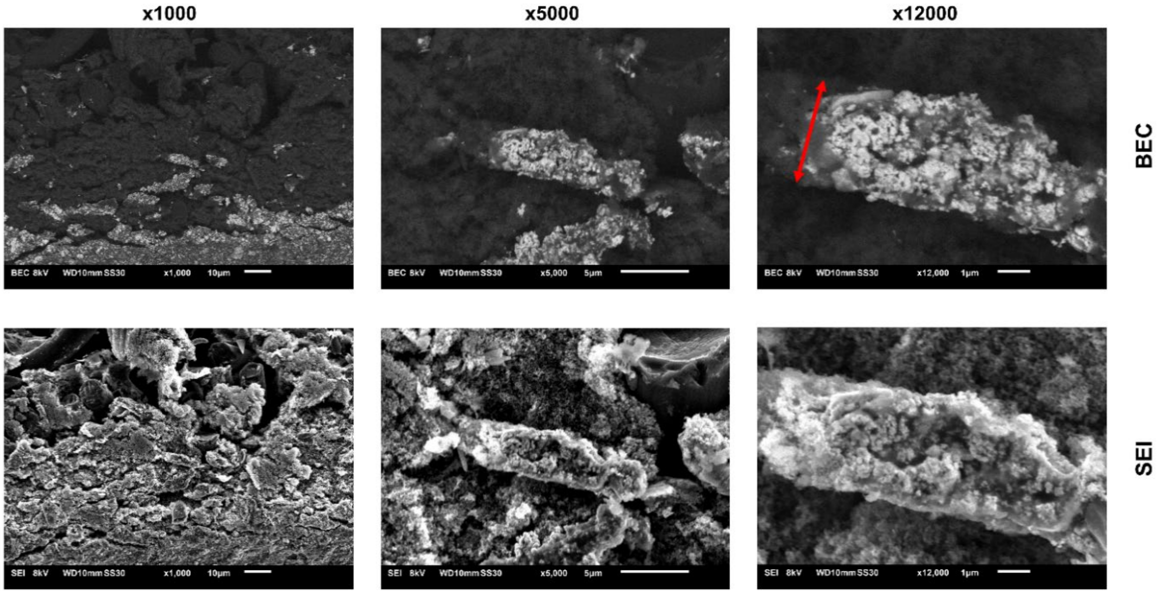

Figure S4: Estimation of catalyst layer (CL) thickness from the fragment lying on its side and facing the detector (top right). Average thickness at 5 random locations of this fragment: 3.5 ± 0.2 µm.

Catalyst primary particle and agglomerate size

The BEC images of this CL fragment show that Ag particles are present in form of agglomerates and primary particles (bright spots in **Figure S5**). They are embedded in a Nafion matrix, which appear as a dark grey mist in

the BEC images. To estimate average Ag agglomerate size, we analyzed a sample set of 18 agglomerates from the BEC image with x12000 magnification (top left): The average diameter was 408 ± 248 nm. The diameters were distributed over a wide range from 200 nm – 1200 nm. To estimate the average primary Ag particle size, we analyzed a sample set of 25 agglomerates from the BEC image with x20000 magnification (top middle): The average diameter was 79 ± 17 nm. This estimate is a bit higher than the nanoparticle size given by the supplier (20 – 40 nm).

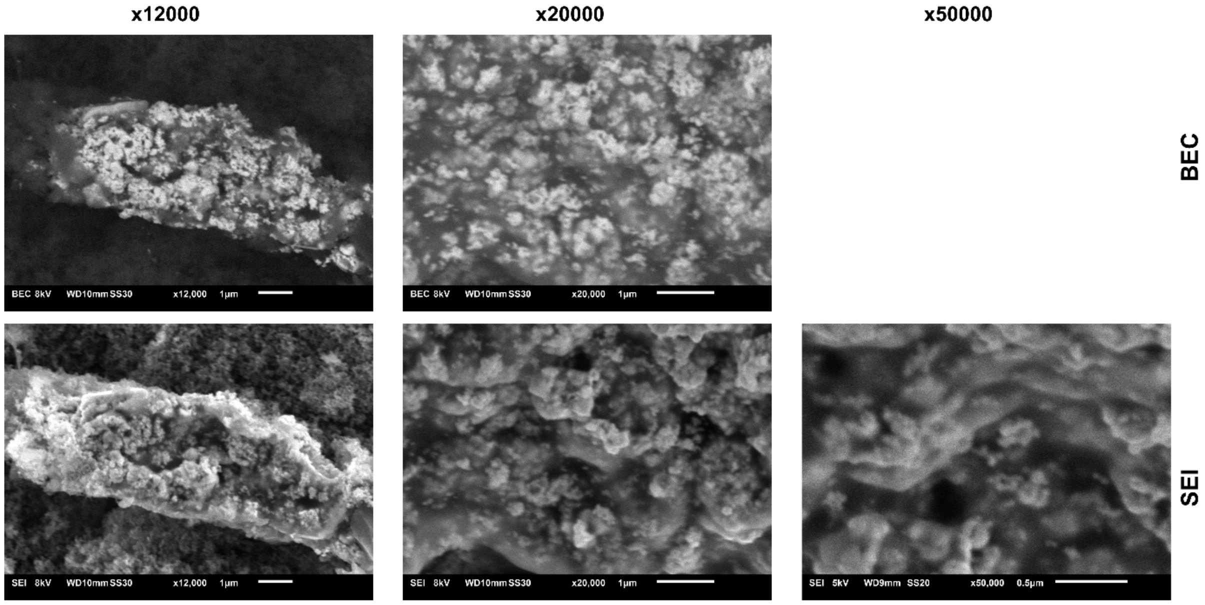

Figure S5: Estimation of primary Ag particles size (79 ± 17 nm) and Ag agglomerates size (408 ± 248 nm) embedded in Nafion.

5. Static contact angle as metric for wettability

A table of the recorded contact angles can be found in the accompanying **Excel file** of the supporting information.

Wetting theory

The wettability or hydrophobicity of the pores in the GDL impacts the breakthrough pressure. For a simple cylindrical pore, the pressure equilibrium between the differential pressure, $\Delta p = p_{\mathrm{L}} - p_{\mathrm{G}}$, and the capillary pressure, p_{C}, is given by the Young-Laplace equation (S1). The surface tension of the liquid is γ in N m^{-1}, the contact angle of the liquid with the pore wall is θ in °, and the pore radius is r in m. Hydrophilic pores (0 < θ < 90°) draw in water and will have liquid breakthrough at any positive pressure. For water to break through hydrophobic pores (90 < θ < 180°) work has to be done to overcome the surface energy.[4]

$$\Delta p = p_L - p_G = p_C = \frac{-2\,\gamma\cos\theta}{r} \qquad \text{(S1)}$$

The wettability is a measure of the ability of a liquid to adhere to a solid material and is a balance of the surface tensions between the three phases (**Figure S6**). From an energy point of view, a system tries to minimize its energy and in this case that means minimizing the surface area between phases with unfavorable high surface tensions (γ in J m^{-2}). The surface tension can also be seen as a force per length (γ in N m^{-1}). That is, a force balance at the three phase interface explains the degree of wetting. The wettability of a material can be measured through the contact angle at this three phase interface. The contact angle, θ, is a result of the surface tension balance and is described by Young's law (S2):[5]

$$\gamma_{\mathrm{lg}}\cos\theta = \gamma_{\mathrm{sg}} - \gamma_{\mathrm{sl}} \qquad \text{(S2)}$$

where γ_{sg} is the solid-gas surface tension, γ_{sl} is the solid-liquid surface tension, and γ_{lg} is the liquid-gas surface tension.

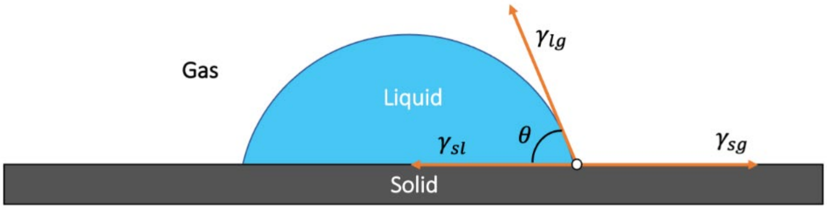

Figure S6: Static contact angle as a result of the force balance at the soli-liquid interface, following Young's law (S2). Here, θ is the contact angle, γ_{sg} is the solid-gas surface tension, γ_{sl} is the solid-liquid surface tension, and γ_{lg} is the liquid-gas surface tension.

Again, GDL materials are slightly more complex due to their surface roughness and chemical heterogeneity. A GDL is often treated with a hydrophobic agent to increase hydrophobicity. Still, the material can contain a few hydrophilic pore voids.[6]

The effect of chemical heterogeneity on the wetting properties can be described with the empirical Cassie-Baxter model (S3). The overall contact angle, θ^*, is determined by the fractions of surface area, f_1 and f_2, belonging to θ_1 and θ_2. The Cassie-Baxter model can also be applied to the effect of surface roughness, by assuming that air bubbles are entrapped inside the surface grooves. Thus, the liquid experiences a composite surface of the solid and the entrapped gas. This model allows for a rough surface to become more hydrophobic, even when the solid's intrinsic contact angle is lower than 90°.[5]

$$\cos\theta^* = f_1 \cdot \cos\theta_1 + f_2 \cdot \cos\theta_2 \quad \text{(S3)}$$

The surface properties of GDLs have been examined more extensively by other authors.[7-10] For example, Gurau *et al.* used the Washburn method to determine the internal contact angle.[7] Gostik *et al.* used the method of standard porosimetry the determine the volume of hydrophilic pores inside the GDL pore network.[6]

Static contact angle measurements

In our study, we measured the external, static contact angle according to the sessile drop technique to assess the wettability of our samples (**Figure S7**). We note that the rough surfaces also lead to the phenomenon of contact angle hysteresis, which would make a dynamic measurement with advancing and receding contact angle more appropriate. Further, this method does not measure internal surface properties, which should be most determining for the saturation characteristics of the GDEs. However, because the measurement of external, static contact angles provides a simple and convenient metric for the wettability, it has been used to study GDL characteristics in the past.[11, 12]

The sample was placed on the sample support (**Figure S7**). We used a pipette to deposit a 10 µL water droplet on a random location of the surface. Then we recorded an image with the digital camera using the ThorCam software. This process was repeated five times per sample. The images were analyzed with ImageJ and the Contact angle plugin. The contact angle was determined by marking the outline of the interface and determining the elliptical contact angle with the manual point procedure function (**Figure S8**). An average angle elliptical angle, θ_{E}, was calculated from the angles $\theta_{\mathrm{E,L}}$ and $\theta_{\mathrm{E,R}}$. These angles arise at intersections of the left and right tangent lines to the ellipse with the solid interface line. The contact angle is then calculated as $\theta = 180° - \theta_{\mathrm{E}}$.

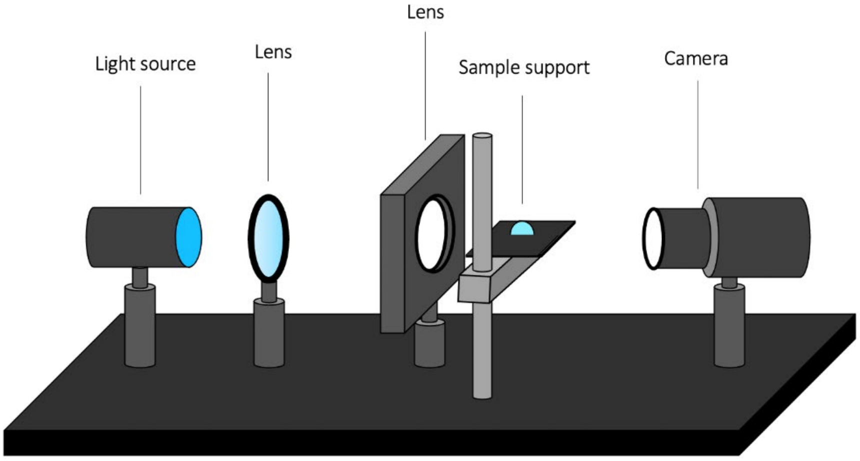

Figure S7: Static contact angle setup used to estimate the wettability of the GDE layers. The setup consist of a light source, two lenses, a sample support and a camera controlled by Thorcam.

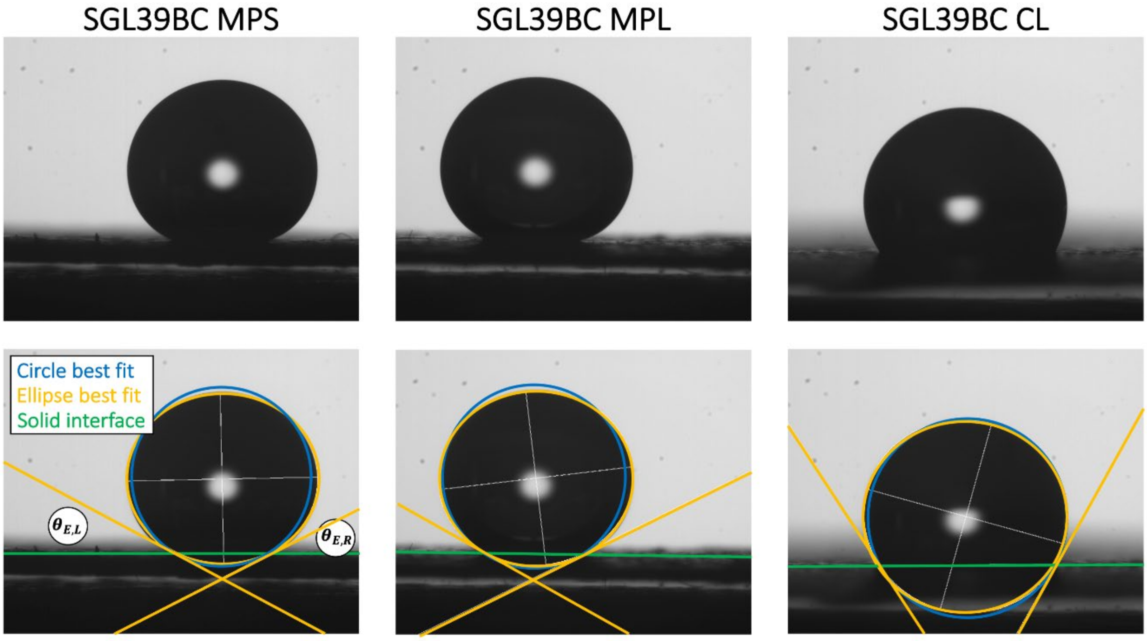

Figure S8: Static contact angle data processing example with SGL 39BC. The top images are the unprocessed images The bottom images were processed using the contact angle function of ImageJ. The angles $\theta_{\mathrm{E,L}}$ and $\theta_{\mathrm{E,R}}$ arise at the intersection between the solid interface line and tangent of the ellipse.

Analyzed samples

We measured contact angles of the CFS and the MPL for each commercial GDL substrate. After applying the CL with the airbrush coater, we also analyzed the contact angles of the CL. To measure the static contact angle of the GDE samples after being used in CO_2 electrolysis, the samples were rinsed with water directly after the electrolysis experiment and left to dry in air for at least 2 days.

Post-electrolysis results

The run times for each sample are specified in the “FE_CO” sheet of the **Excel file**. We observed a reduction in CL contact angle for all samples. This can be explained by the reduction of AgO to Ag by applying a negative potential. Probably this change occurs within seconds are minutes after applying a potential.

The change in contact angle for the CFS due to electrolysis is not significant for all GDL samples except for H23C6. For H23C6, we carried out the experiment at 100 mA cm^{-2}. After a run time of 30 min, θ_{CFS} changed by –25 ± 9°. A similar current-dependent degradation rate was also reported by Leonard *et al.*.[13]

6. Flooding resistance

The uncoated GDLs were characterized for their liquid breakthrough pressure in a 2-compartment cell prior to the electrolysis experiments. The flow-by pressure window, Δp^*, of the coated GDE samples was determined right before the electrolysis experiments were carried out in the 3-compartment cell.

H_2O breakthrough pressure for uncoated GDLs (CFS + MPL)

The measurement configuration to determine the liquid breakthrough pressure, Δp_{L}^*, with water is shown in **Figure S9 a**. We installed the uncoated GDL sample (CFS + MPL) in the characterization cell (**Figure S10**). The MPL was facing the liquid compartment of the cell leaving a cross-sectional area of 3.8 cm^2 exposed. The sample was placed on a polypropylene mesh for mechanical support **Figure S9 c**. We set the liquid flow rate of the peristaltic pump to 1 mL min^{-1} to slowly fill and pressurize the liquid compartment for 15 min. The water was forced through the GDL because the outlet of the liquid compartment was closed. The breakthrough pressure, Δp_{L}^*, was determined by observing at which pressure the first droplet appeared at the GDL surface **Figure S9 b**. We used a mass flow controller (MFC) to supply 30 mLn min^{-1} of CO_2 gas through the gas inlet to remove permeated water from the gas compartment. The gas and the liquid left the cell together through the gas outlet. The pressure gradient across the sample was recorded with a Deltabar S differential pressure meter (Endress+Hauser, Switzerland). We performed these measurement with one sample per GDL model. For the SGL 39BC, we carried out one repeat experiment to estimate the random error.

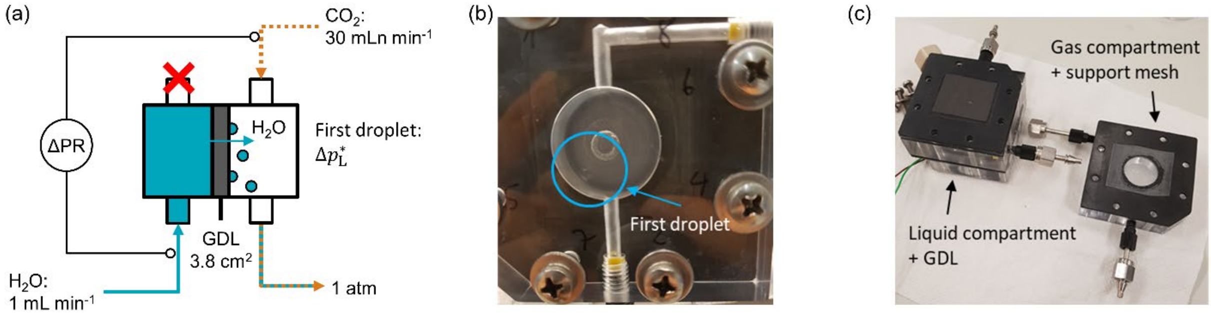

Figure S9: Experimental overview for liquid breakthrough measurements for uncoated GDL samples. **(a)**: Measurement configuration for H_2O breakthrough pressure Δp_{L}^*. **(b)**: Visual determination of liquid breakthrough pressure: The differential pressure was recorded when the first liquid droplet became visible on the GDL surface in the gas compartment. **(c)**: Disassembled characterization cell with GDL sample on top of liquid compartment and support mesh on gas compartment.

Our recorded H_2O breakthrough pressures for the uncoated GDL (CFS + MPL) samples are listed **Table S3**. We analyzed a second sample of SGL 39BC to get an estimate for the variability of the results. The two resulting values of 57 mbar and 67 mbar give an average breakthrough pressure of 62 mbar with a sample standard deviation of ±7 mbar. Because it was not feasible to carry out a large number of repeats for every GDL model, we estimate the random error based on similar studies we found in literature. A thorough study on liquid breakthrough pressure through polymer electrolyte fuel cell (PEFC) GDLs determined an error of about ±10 mbar for Toray papers.[14] We use this value as an estimate for all GDL models that have their breakthrough pressure in a similar order of magnitude (Toray paper, SGL paper, ELAT paper). For the Freudenberg H23C6, we use an estimate of ±100 mbar based on the study by Leonard et al.[13] We did not record the gas breakthrough pressure, Δp_{G}^*, for the uncoated samples, but we assume that this value is 0 mbar for all GDL types.

Table S3: Experimentally determined H_2O breakthrough pressure, Δp_L^*, and estimated standard error, $\sigma_{\Delta p_L^*}$, in mbar.

Material	TGP-H-060	TGP-H-090	TGP-H-120	SGL 22BB	SGL 39BC	LT1400W	H23C6
Sample 1	65	77	64	44	57	52	464
Sample 2	n.a.	n.a.	n.a.	n.a.	67	n.a.	n.a.
Mean Δp_L^*	65	77	64	44	62	52	464
$\sigma_{\Delta p_L^*}$	±10	±10	±10	±10	±10	±10	±100

Flow-by pressure window Δp^* of the coated GDE

We measured the flow-by pressure window, Δp^*, of the GDE right before the CO_2 electrolysis experiments. For this purpose, the GDE was installed in the 3-compartment cell (**Figure S13**) and integrated into the electrolysis setup (**Figure S16**). The electrolyte reservoir was filled with 1 M $KHCO_3$ saturated with CO_2 and the liquid lines were primed.

The pump was set to a liquid flow rate of 100 mL min^{-1} for each electrolyte channel. In its initial state, the reactor exhibited the breakthrough of feed gas because of the backpressure provided by the check valve at the outlet (345 mbar). The liquid back pressure was then slowly increased in increments of 10 mbar until a transition to the next flow regime occurred. The corresponding differential pressure Δp was noted. We recorded the transition between the following regimes:

- Gas breakthrough (Flow-through)
- No breakthrough (Flow-by)
- Liquid breakthrough

After these measurements, the liquid pressure was again reduced until gas breakthrough occurred. We used separate samples of the Freudenberg H23C6 for each of the experiments (Flow-by pressure window, 100 mA cm^{-2}). For all other GDLs, the same sample was used for all these experiments.

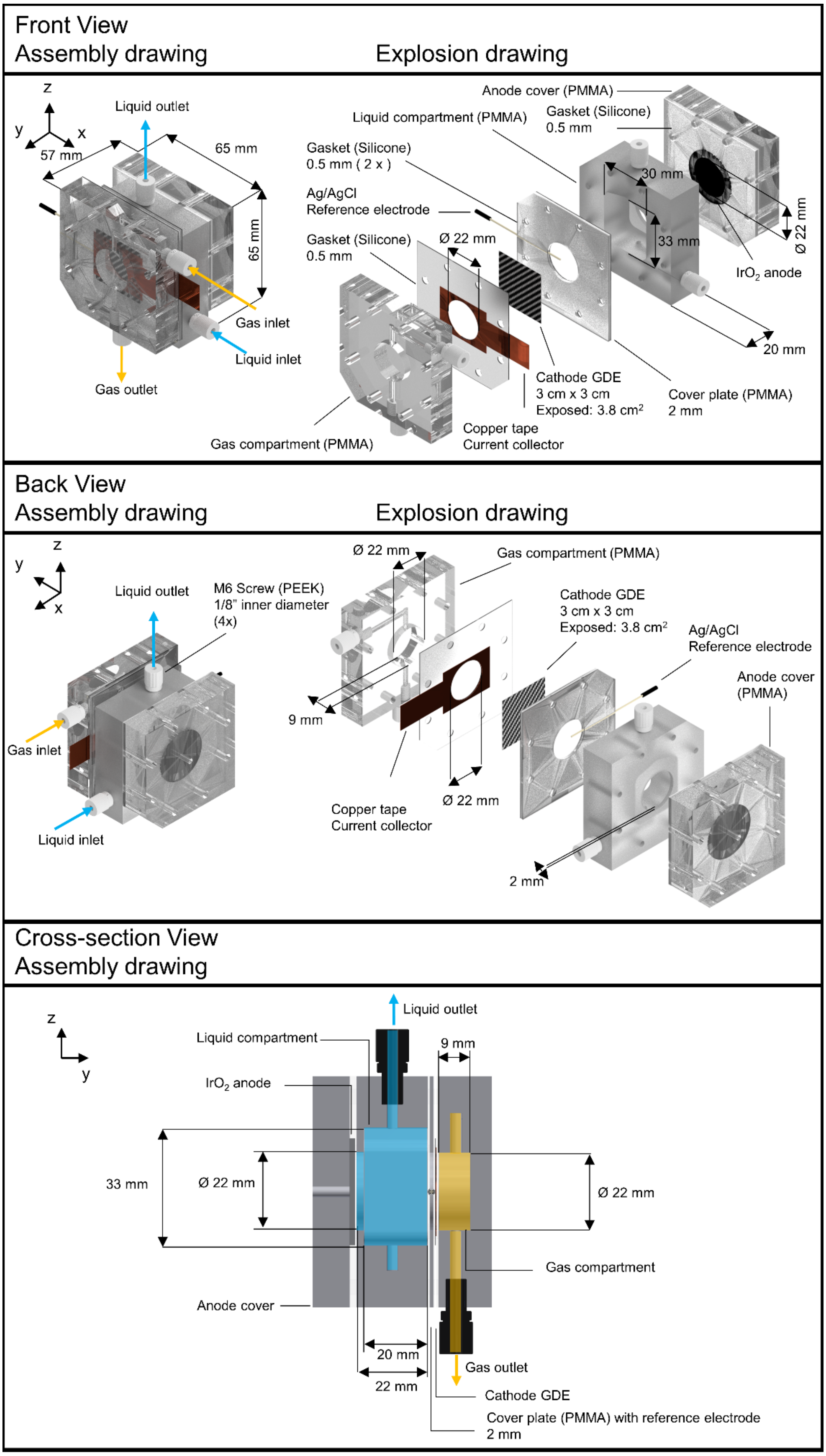

Figure S10: Schematic of the 2-compartment characterization cell used to determine the H_2O breakthrough pressure, Δp_L^*, the CO_2 permeability constant, P_{CO_2}, and the limiting overall O_2 mass transfer coefficient, k_{O_2}.

7. CO_2 Permeability for uncoated GDL (CFS + MPL)

The CO_2 permeability was characterized with the 2-compartment flow cell (**Figure S10**) using the configuration shown in **Figure S11**. The gas was forced through the GDL from the CFS side by closing the gas and liquid outlet. We varied the gas flow rate from 0.1 to 1.4 L min^{-1} (20°C) in steps of 0.1 L min^{-1}. Each flow rate step was held for 90 s to allow the system to reach a steady state. For the sample Freudenberg H23C6, the flow rate interval was 10 to 100 mL min^{-1} (20°C) in steps of 10 mL min^{-1}. The pressure drop of the empty cell and the tubing was recorded and subtracted from the pressure drops recorded for each sample.

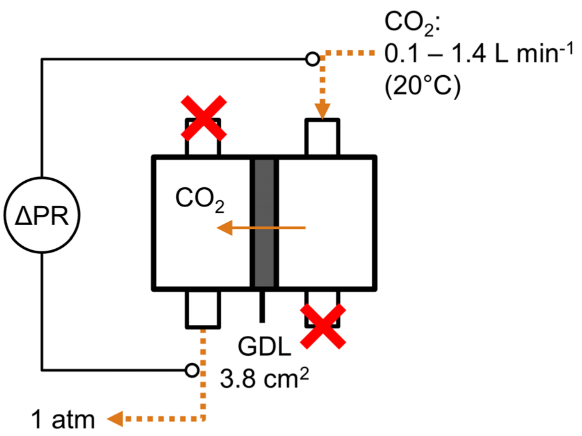

Figure S11: Configuration of 2-compartment characterization flow cell used to measure the CO_2 permeability.

We determined the permeability constant, $P_{\mathrm{CO_2}}$, in mL min- mbar^{-1} by plotting the volumetric flow rate, F, through the cross-sectional area, A, against the pressure drop across the sample, Δp, according to Darcy's law (S4).[15] The sample thickness, δ, the dynamic viscosity, μ, the permeability, P' in m^2, and A are incorporated into the empirical permeability constant, $P_{\mathrm{CO_2}}$.

$$F = \frac{P'}{\delta_{\mathrm{CFS+MPL}}} \cdot \frac{A}{\mu} \cdot \Delta p = P_{\mathrm{CO_2}} \cdot \Delta p \qquad \text{(S4)}$$

The resulting permeability curves are displayed in **Figure S12**. The permeability constant, $P_{\mathrm{CO_2}}$, was determined from the slope of the curve and is listed in **Table S4**. The samples TGP-H-060, TGP-H-090, TGP-H-120, and SGL 22BB show a very similar pressure drop behavior, which is close to the 1 mbar detection limit our equipment. Therefore, we can not distinguish the permeability constants of these samples and only give an estimate. In comparison to these samples, SGL 39BC and ELAT LT1400W have a lower permeability. We estimate a relative error of ±15% based on the resolution (1 mbar) relative to the measured range (6 mbar). In contrast, the slope of the Freudenberg H23C6 is much lower and the data is a lot less noisy. We estimated a relative error of ±5%.

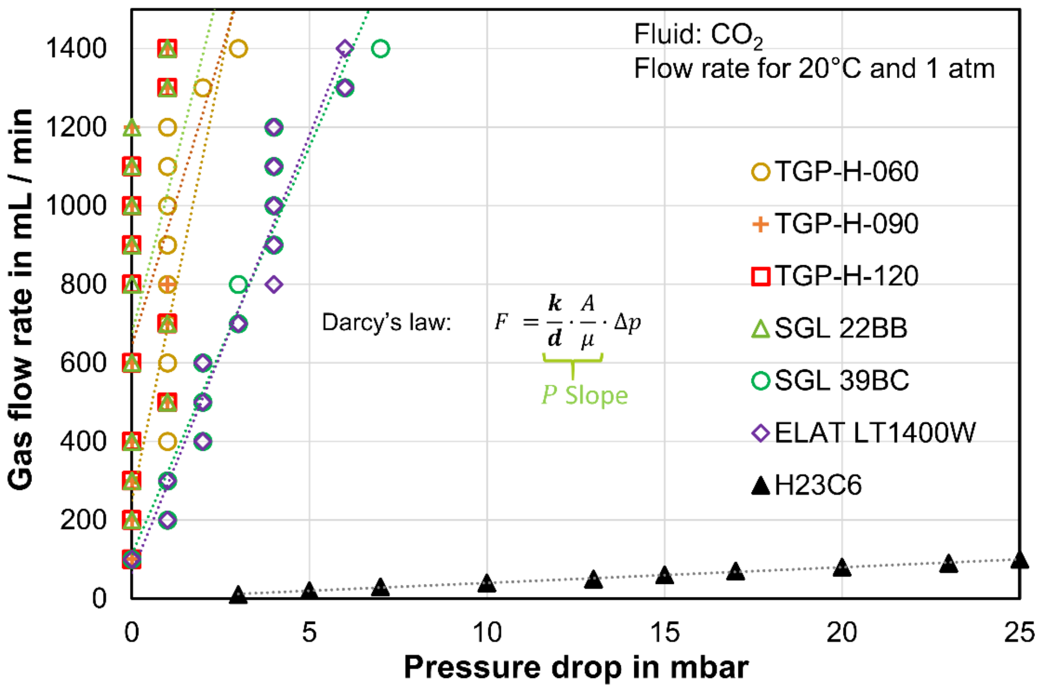

Figure S12: CO_2 permeability plots for uncoated GDL samples. The permeability constant, P, is derived from the slope.

Table S4: Experimentally determined CO_2 permeability constants, P_{CO_2}, in mL min^{-1} mbar^{-1}.

Material	TGP-H-060	TGP-H-090	TGP-H-120	SGL 22BB	SGL 39BC	LT1400W	H23C6
P_{CO_2}	>300	>300	>300	>300	206	226	4

8. CO_2 electrolysis procedure

The CO_2 mass transfer of the GDE materials was characterized with a 3-compartment electrolysis cell, which was integrated into an automated experimental setup.

8.1 Assembly of 3-compartment CO_2 electrolysis cell

The front view of the 3-compartment electrolysis cell is shown in **Figure S13**. The transparent body of the cell was machined out of poly methyl methacrylate (PMMA) by our workshop. This allowed the observation of the flow regime at the GDE during operation. The cell was connected to tubing with fittings made of poly ether ether ketone (PEEK), which were supplied by the IDEX corporation (Illinois, USA). The PMMA parts were sealed against each other with 0.5 mm silicone gasket (Eriks, Netherlands). The cation exchange membrane (Selemion CMV) was sandwiched between two gaskets. The differential pressure between the gas compartment and the catholyte compartment was measured directly inside the cell. For this purpose, the differential pressure meter was attached to the 1/16” outer diameter tubes, which were directly connected to the inside of the cell (**Figure S13**).

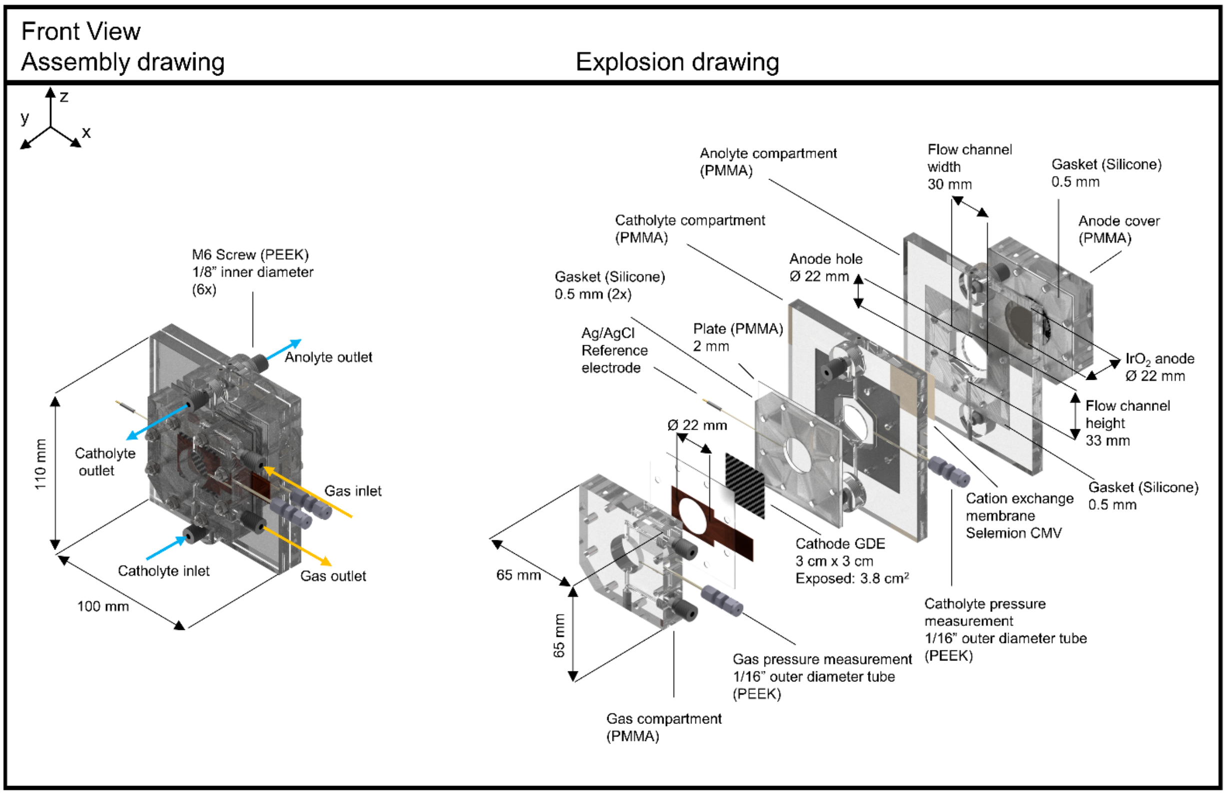

Figure S13: Front view of the 3-compartment electrolysis cell used to determine the flow-by pressure window pressure, $\Delta p_{\mathrm{L}}^{*}$, at open circuit potential and the faradaic efficiency for CO, FE_{CO}, as a function of current density. PEEK stands for poly ether ether ketone. PMMA stands for poly methyl methacrylate.

The back view of the 3-compartment electrolysis cell is shown in **Figure S14**. The GDE was connected to the potentiostat with a copper tape (Conrad, Netherlands), which served as current collector. We integrated an Ag/AgCl micro-reference electrode (Type: Leak free reference electrode; Supplier: Multi Channel Systems, Germany) into the center of the PMMA sheet that was next to the cathode GDE. The gap between cathode and tip of the reference electrode was 0.5 mm. The anode plate electrode was made of a titanium disk coated with an IrO_2 oxygen evolution catalyst (Magneto Special Anodes, Netherlands). We connected it to the potentiostat with a wire running to the back side of the disk through a hole in the anode cover.

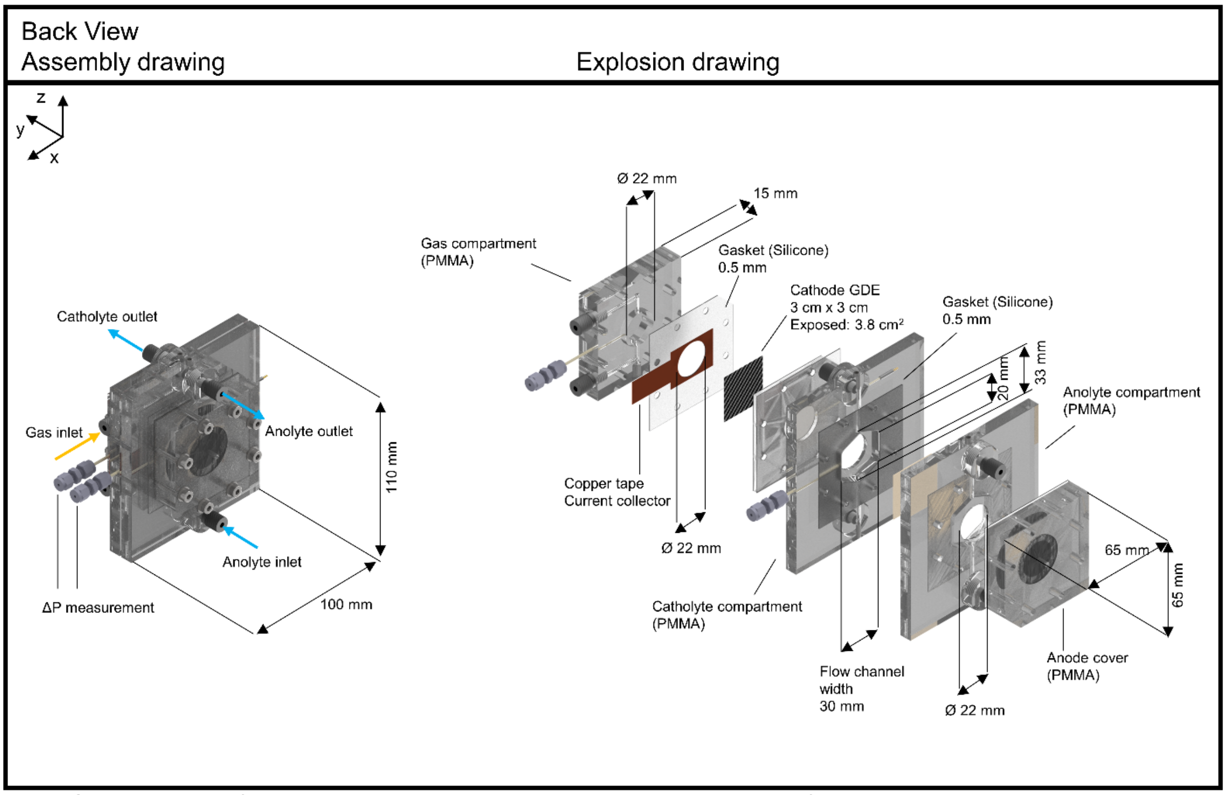

Figure S14: Back view of the 3-compartment electrolysis cell used to determine the flow-by pressure window pressure, Δp_L^*, at open circuit potential and the Faradaic efficiency for CO, FE_{CO}, as a function of current density. PMMA stands for poly methyl methacrylate.

Figure S15 shows the cross-section of the electrolysis flow cell. We choose and upward flow direction for the electrolytes to facilitate the removal of gas bubbles. The gas flows downward to remove any liquid from the gas compartment.

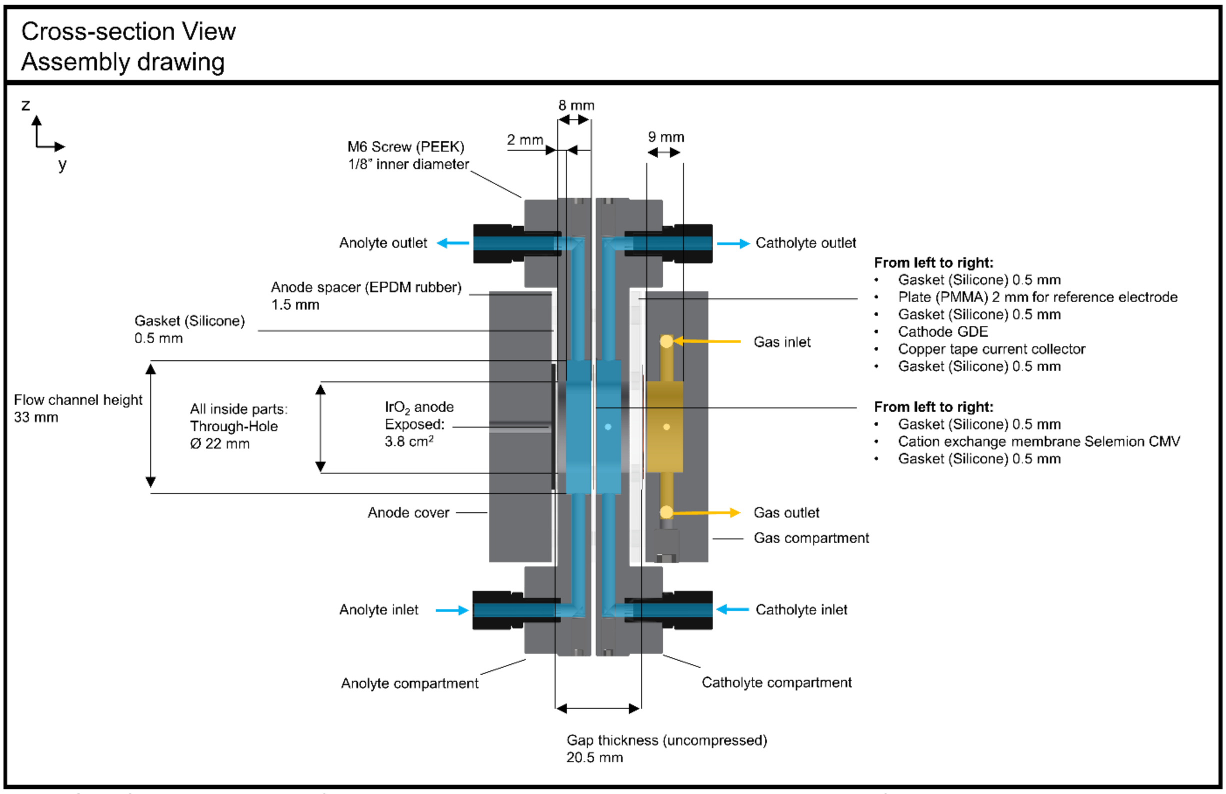

Figure S15: Cross-section view of the 3-compartment electrolysis cell used to determine the flow-by pressure window pressure, Δp_L^*, at open circuit potential and the Faradaic efficiency for CO, FE_{CO}, as a function of current density.

8.2 Operation of the CO_2 electrolysis setup

The CO_2 reduction experiments were carried out with the electrolysis setup shown in **Figure S16** and **Figure S17**. We used Labview (Version 2018, National Instruments) to record online data of the various sensors and to control the pump and the electronic valves.

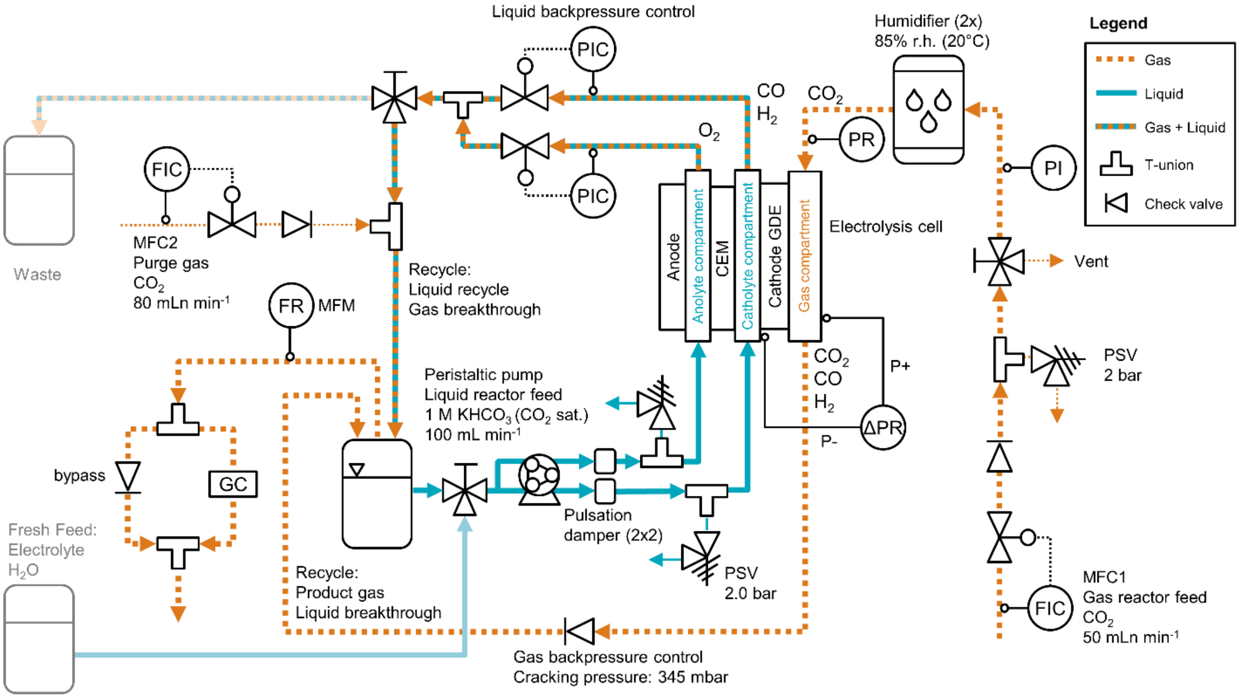

Figure S16: Extended process flow diagram for CO_2 electrolysis setup with different pressure control. The gas flow rates were controlled with mass flow controllers (MFC). Check valves were used to prevent the backflow of liquid into the MFCs. Pressure safety valves (PSV) were installed in line to prevent the unexpected buildup of pressure. The gas feed pressure was measured with an analog pressure indicator (PI) and recorded after the humidifiers (PR). The differential pressure between the gas and the catholyte compartment were recorded with a differential pressure meter (ΔPR). The anolyte and catholyte compartment were separated with a cation exchange membrane (CEM). The backpressure of both electrolyte streams was controlled (PIC) before both streams were combined and recirculated. The product gases were collected from all process streams and combined in the head space of the electrolyte reservoir. Their flow rate was recorded (FR) with a mass flow meter (MFM) and the composition analyzed with a gas chromatography system (GC) to calculate the Faradaic efficiency.

Gas feed flow path

The CO_2 feed gas was supplied from a CO_2 cylinder. The gas flow rate was controlled and measured with a mass flow controllers (MFC1) of the type F-201CV-500 from Bronkhorst (Netherlands). We passed the gas through two custom-made bubble columns (**Figure S18** and **Figure S19**) in series to humidify the feed with water. The temperature and relative humidity of the gas feed was recorded after the humidification stage with a humidity sensor (Type: HC2A-S Hygroclip RV+T sensor; Supplier: Acin Instrumenten, Netherlands). The pressure of the gas feed was recorded with a Deltabar S pressure meter (Endress+Hauser, Switzerland). We used another Deltabar S to record the pressure difference between the gas compartment (positive terminal: P+) and the liquid compartment (negative terminal: P-). The backpressure of the gas outlet was set by a SS-CHS2-5 check valve (Swagelok, Netherlands) with a nominal cracking pressure of 345 mbar.

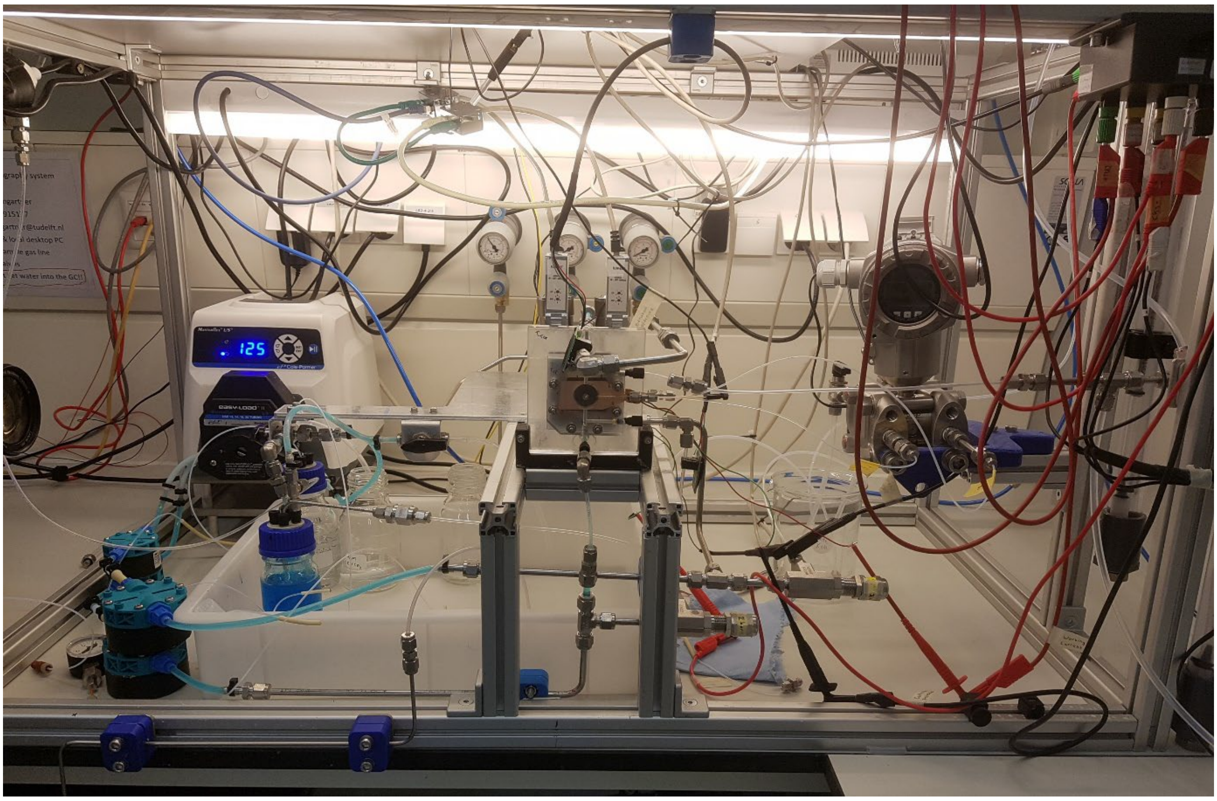

Figure S17: Picture of CO_2 electrolysis setup. The peristaltic pump is located on the left. The 3-compartment flow cell is in the center of the image.

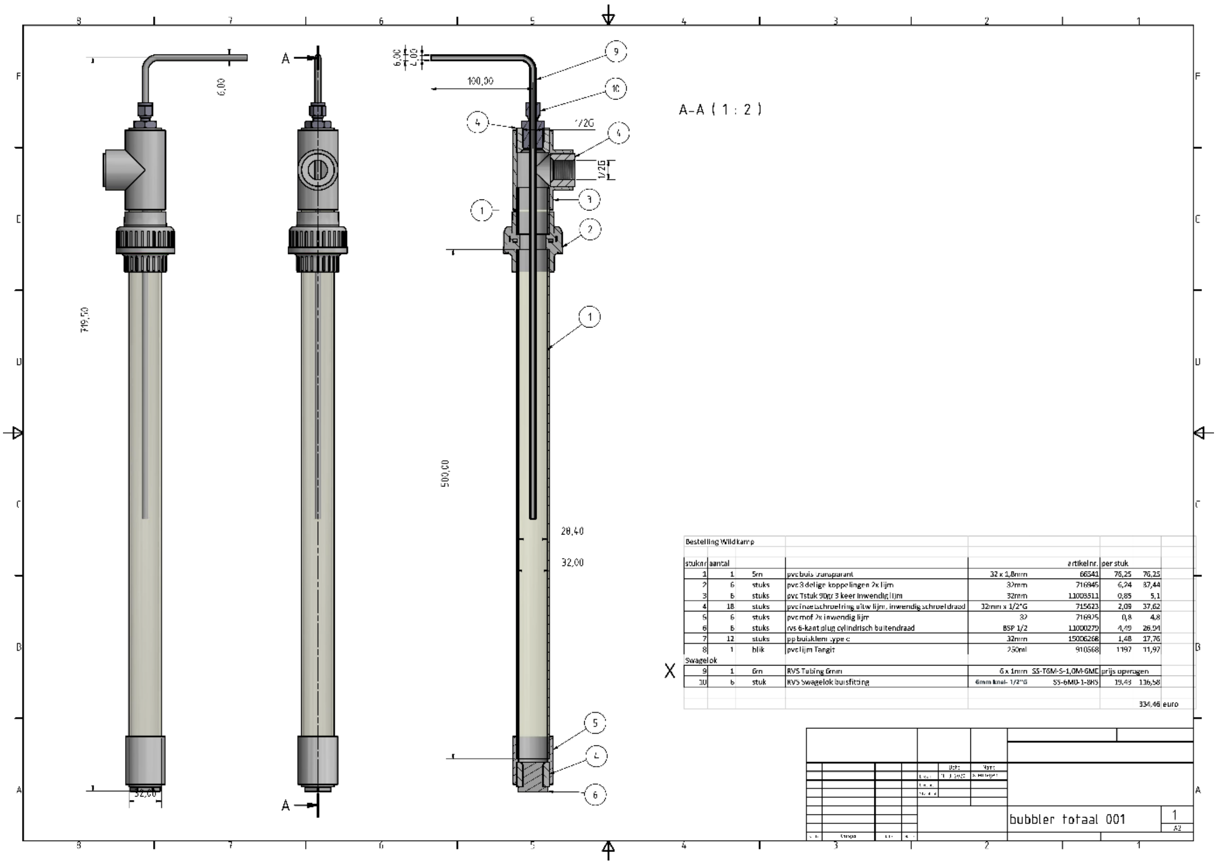

Figure S18: Technical drawing of the humidifier column. The units are in mm.

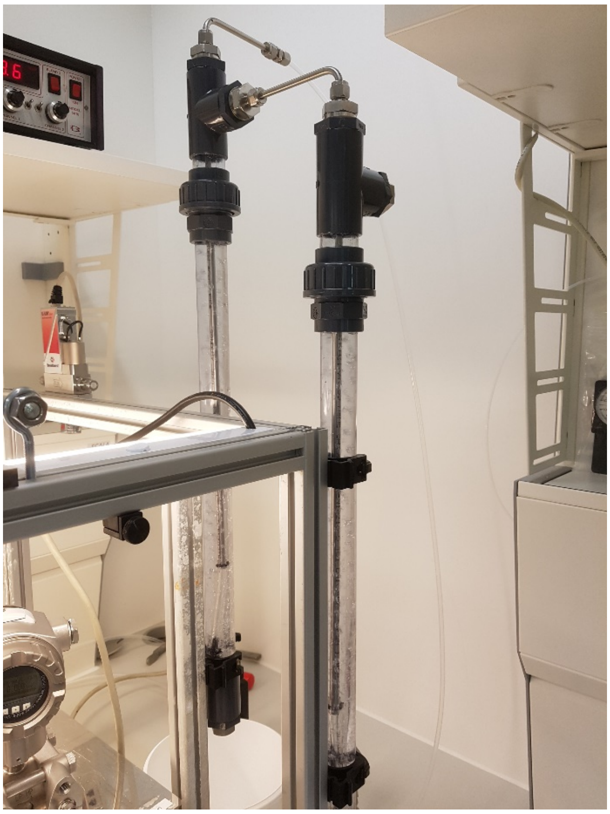

Figure S19: Two custom-made humidifier columns made from PVC pipes were used to humidify the CO_2 feed to 85 % relative humidity (r.h.) at 20°C.

Electrolyte flow path

The 1 M $KHCO_3$ electrolyte saturated with CO_2 was prepared by diluting concentrated KOH (50 wt%, analytical grade, Alfa Aesar) to 1 M KOH. The CO_2 was bubbled through the solution to until the pH value was stable. The bulk pH of the electrolyte was measured prior to the experiments and is listed in the accompanying **Excel file**. The liquid lines and reactor was flushed before every experimental run. The electrolyte reservoir and liquid lines were filled with 100 mL fresh electrolyte. We used a peristaltic pump (Type: Masterflex L/S peristaltic pump; Supplier: Cole Parmer) to recirculate the electrolyte through the reactor and the liquid lines with a flow rate of 100 mL min^{-1} for the catholyte channel and for the anolyte channel each. Two pulsation dampers (Types: FPD 1.06, FPD 1.10; Supplier: KNF, Switzerland) reduced the pressure fluctuations caused by the pump. We controlled the liquid back pressure of both channels with electronic control valves (Type: P-502C-6K0R; Supplier: Bronkhorst, Netherlands). After the experimental run, we collected a sample of the electrolyte to measure the formic acid content with HPLC. In addition to CO and H_2, formic acid has also been reported to form on Ag catalysts in small amounts.[16]

Product gas flow path

Unreacted CO_2 and product gases left the reactor through the gas outlet and entered the head space of the electrolyte reservoir. Product gases forming on the catholyte side (CO, H_2) and the anode side (O_2) were carried out of the reactor by the electrolyte stream. After the two electrolyte streams were recombined, we added a CO_2 purge gas stream to facilitate the transfer of product gases into the gas phase. The CO_2 purge gas stream further ensured that the electrolyte remained saturated with CO_2 during the experimental run. All the product gases were collected in the headspace of the electrolyte reservoir and passed through a mass flow meter (MFM) to record the flow rate (Type: F-111B-500; Supplier: Bronkhorst, Netherlands). The gas composition was analyzed with a gas chromatography system (Type: Compact GC 4.0; Supplier: Interscience, Netherlands).

Calculation of Faradaic efficiency

The Faradaic efficiency of gas species FE_i (H_2, CO) was calculated with the recorded current, I, Faraday's constant, F, the stoichiometric number of electrons exchanged, z_i ($z_i = 2$ for H_2 and CO), the corrected MFM gas flux, $\dot{N}_{\mathrm{MFM}}$, and the gas concentration, C_i ,using (S5).

$$FE_i = \frac{z_i \cdot F \cdot C_i \cdot \dot{N}_{\mathrm{MFM}}}{I} \quad \text{(S5)}$$

The simple gas conversion factors provided by the supplier of the MFM are listed in **Table S5**. We did not detect the components CH_4 or C_2H_4 in any of our product gas samples. Therefore, CO_2 is the only component that differs significantly from a conversion factor of 1 in our product mixture. This allows us to simplify the calculation of the mixture conversion factor K_{mix}.

Table S5: Single component gas conversion factors K_{i} for 20°C and 1 atm provided by Bronkhorst General Manual Digital Instruments.

Component	K_{i}
**H_2**	1.01
**N_2**	1.00
**O_2**	0.98
**CO_2**	0.74
**CO**	1.00
**CH_4**	0.76
**C_2H_4**	0.60

We assume that the components N_2, H_2, O_2, are equivalent in their conversion factor to CO. We then developed a simple linear model using the Fluidat flow calculation tool (Bronkhorst, Netherlands). This tool allows to calculate K_{mix}, which converts the recorded gas flow, $\dot{N}_{\mathrm{MFM,nominal}}$, (MFM calibrated for 90 vol% CO_2, 5 vol% CO, 5 vol% H_2 at 10 bar (a) and 20°C) to the corrected gas flow (actual product mixture at 0.1 bar (g) and 20°C). The corrected MFM gas flux, $\dot{N}_{\mathrm{MFM}}$, is then calculated with (S6). Our model assumes the product gas mixture is a two component mixture made up of CO_2 and CO (**Figure S20**).

$$\dot{N}_{\mathrm{MFM}} = \dot{N}_{\mathrm{MFM,nominal}} \cdot K_{\mathrm{mix}} \quad \text{(S6)}$$

The mixture conversion factor, K_{mix}, is calculated with the regression formula (S7) determined in **Figure S20**.

$$K_{\mathrm{mix}} = 1.291 - 0.2764 \cdot \frac{C_{\mathrm{CO_2}}}{[\mathrm{vol\%}]} \quad \text{(S7)}$$

We calculated the volumetric concentration of CO_2, $C_{\mathrm{CO_2}}$, in vol % with (S8).

$$C_{\mathrm{CO_2}} = 100\ \mathrm{vol\%} - C_{\mathrm{CO}} - C_{\mathrm{H_2}} - C_{\mathrm{O_2}} - C_{\mathrm{N_2}} \quad \text{(S8)}$$

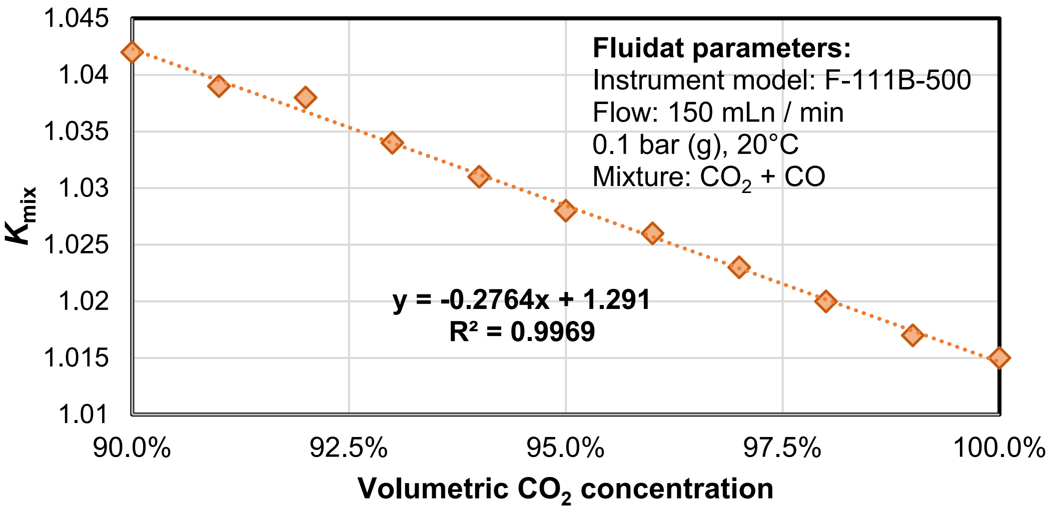

Figure S20: Determination of the mixture conversion factor, K_{mix}: The linear regression model to calculate K_{mix} is based on data points calculated with the Fluidat flow calculation tool (Bronkhorst, Netherlands). The gas mixture consists of CO_2 and CO.

The Faradaic efficiency values of the product gases CO and H_2 are listed in the accompanying **Excel file** in the sheet "FE" for all experiments.

We can estimate the Faradaic efficiency of formic acid, FE_{HCOOH}, with (S9). We collected a sample of the electrolyte after electrolysis and analyzed it with HPLC to determine the concentration of formic acid, c_{HCOOH}, in mol L^{-1}. The total passed charge, Q, was recorded by the potentiostat. The electrolyte volume $V_{\mathrm{electrolyte}}$ was about 60 mL for each experiment.

$$FE_{\mathrm{HCOOH}} = \frac{2 \cdot F \cdot c_{\mathrm{HCOOH}} \cdot V_{\mathrm{electrolyte}}}{Q} \tag{S9}$$

The detailed values for all GDL samples are listed in the accompanying **Excel file** for all experiments. We calculated a FE_{HCOOH} between 2% to 4% for our different materials samples. This roughly makes up the amount to complete the Faradaic efficiencies of H_2 and CO to 100%.

9. Overall O_2 mass transfer coefficient k_{O_2} of GDE

Experimental setup

The overall O_2 mass transfer coefficient, k_{O_2}, was measured electrochemically with the configuration shown in **Figure S21.** At sufficiently high currents, the reduction at the cathode GDE becomes limited by the O_2 mass transfer through the GDE (Cathode reaction: $O_2 + 2\ H_2O + 4\ e^- \rightarrow 4\ OH^-$). We placed the coated GDE samples (CFS + MPL + CL) in the characterization cell (**Figure S10**). The MPL+CL was facing the liquid compartment of the cell leaving a cross-sectional area of 3.8 cm^2 exposed. A titanium plate with a circular hole was used as a current collector. The plate was inserted between GDE and gas compartment. The Ag/AgCl micro-reference electrode was placed at a distance of 1 mm from the cathode surface. A Nickel plate with the same area as the cathode was used as counter electrode (Anode reaction: $4\ OH^- \rightarrow O_2 + 2\ H_2O + 4\ e^-$).

Experimental procedure

During electrolysis measurements, the electrolyte (6 M KOH for high conductivity) was pumped through the liquid compartment at a flow rate of 20 mL min^{-1} and recirculated. Pressurized air from the house line was supplied at a flow rate of 40 mLn min^{-1} to the gas compartment. The backpressure of the gas outlet was set by a SS-CHS2-5 check valve (Swagelok, Netherlands) with a nominal cracking pressure of 345 mbar. The pressure controller of the liquid lines was used to balance the pressure between the gas and the liquid compartment until no gas or liquid breakthrough was observed. The gas pressure was recorded before starting the electrolysis process.
We recorded a minimum of 3 linear sweep voltammetry (LSV) scans for each sample. The cathode potential was varied from 0 V to -2.0 V vs SHE with a scan rate of 20 mV s^{-1}. We waited for 3 min between scans to give the system time to re-equilibrate. We carried out additional LSV scans with a N_2 gas feed to record the current of the competing hydrogen evolution reaction (Side reaction: $2\ H_2O + 2\ e^- \rightarrow H_2 + 2\ OH^-$).

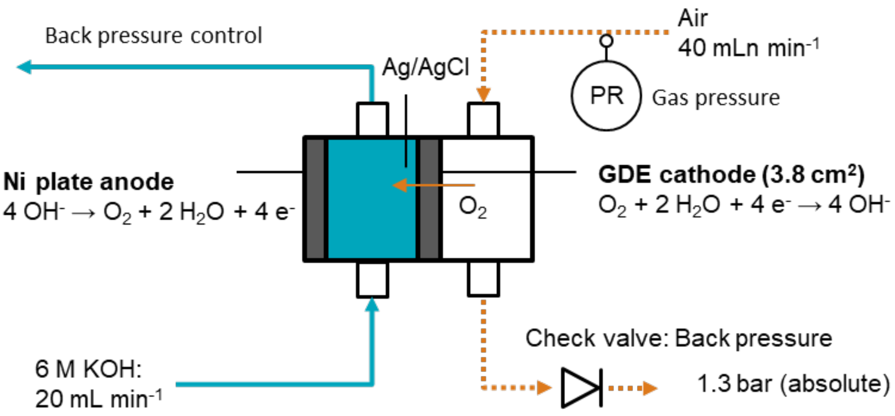

Figure S21: Configuration for limiting O_2 mass transfer measurement. The GDE cathode reduces O_2 at the catalyst surface to OH^- ions. The current is limited by the mass transfer rate of O_2 from the gas bulk through the GDL to the catalyst layer.

Calculation of overall O_2 mass transfer coefficient

To determine the overall O_2 mass transfer coefficient, k_{O_2}, for each GDE, the limiting current density, j_{lim} was extracted from the LSV scans according to **Figure S22**. The data processing parameters are listed in **Table S6**. We excluded the first two scans from our analysis because the recorded current curves drifted during the first scans. We also excluded scans with larger signal fluctuations caused by gas bubbles interfering with the measurement. The data processing is also described in the sheet "kO2" of the accompanying **Excel file**.

We define the potential window of the limiting current plateau with the lower potential limit, $E_{\mathrm{lim,lower}}$, and the upper potential limit, $E_{\mathrm{lim,upper}}$, in V vs SHE. The scan numbers (Scan #) included in current calculations are listed in **Table S6**. For each of these scans, we then determined the average limiting current, j_{lim}, in mA cm^{-2} and its corresponding sample standard deviation, $\sigma_{j_{\mathrm{lim}}}$, to consider the fluctuations in the current. Using j_{lim} and $\sigma_{j_{\mathrm{lim}}}$ of each scan, we then calculated the average limiting current density, $\bar{j}_{\mathrm{lim}}$, and the standard error of the limiting current density, $\bar{\sigma}_{j_{\mathrm{lim}}}$, for the GDE sample.

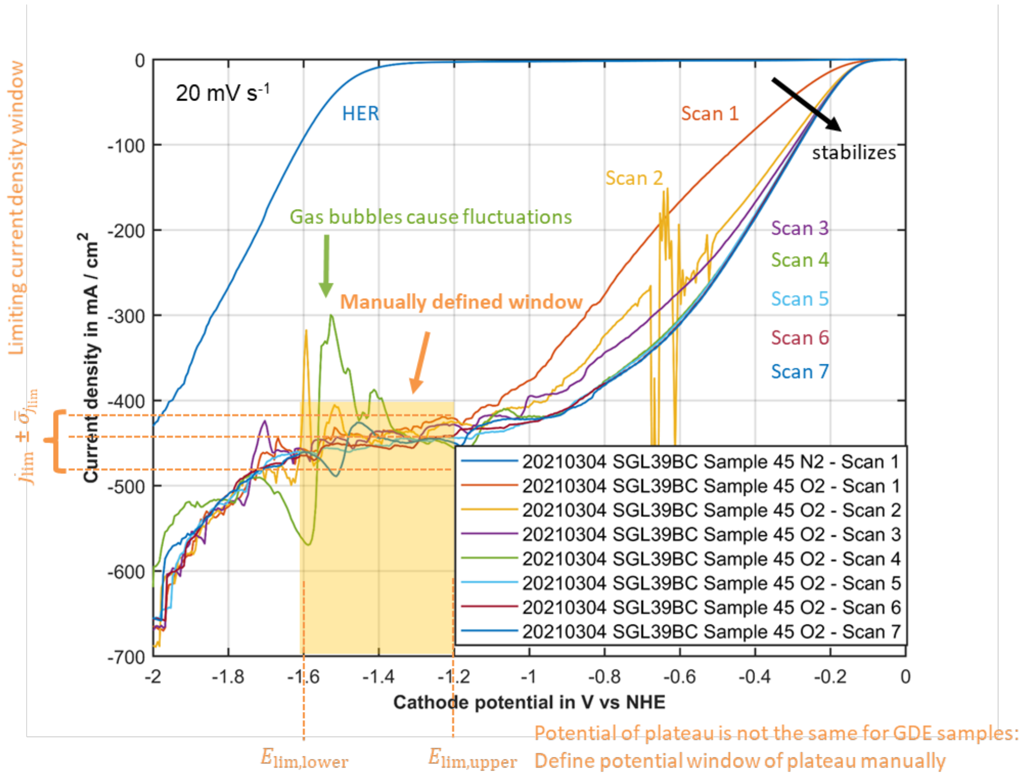

Figure S22: O_2 mass transfer coefficient raw data processing for SGL 39B determined by linear sweep voltammetry (LSV). The O_2 scans 1-7 use an air feed at the cathode for the reduction of O_2 to OH^-. The scan with the N_2 gas feed shows the current of the hydrogen evolution reaction for comparison. The average limiting current density j_{lim} in mA cm^{-2} and the sample standard deviation of the limiting current density $\sigma_{j_{\mathrm{lim}}}$ was calculated for the limiting current plateau in the potential window between $E_{\mathrm{lim,lower}}$ and $E_{\mathrm{lim,upper}}$ in V vs SHE. The first two scans and scans with large fluctuations were not included for calculation of the average limiting current densities.

The limiting O_2 molar flux, $\dot{n}_{\mathrm{O_2,lim}}$, in mol cm^{-2} s^{-1} was calculated from $\bar{j}_{\mathrm{lim}}$ with Faraday's law (S10). Faraday's constant is $F = 96485$ C s^{-1} and the number of electrons exchanged in the reaction is $z = 4$.

$$\dot{n}_{\mathrm{O_2,lim}} = \frac{\bar{j}_{\mathrm{lim}}}{F \cdot z} \tag{S10}$$

We assumed that the limiting O_2 flux, $\dot{n}_{\mathrm{O_2,lim}}$, is proportional to the overall O_2 mass transfer coefficient of the GDE, $k_{\mathrm{O_2}}$ in cm s^{-1}, and the O_2 concentration gradient between the bulk of the gas compartment, $C_{\mathrm{O_2,bulk}}$, and the catalyst surface, $C_{\mathrm{O_2,Cat.}}$. We neglected concentration gradients in flow direction because the convective O_2 flux into the gas compartment was about 56% larger than the O_2 consumed in the reaction. By assuming that the O_2 concentration at the catalyst surface, $C_{\mathrm{O_2,Cat.}}$, dropped to 0 mol cm^{-3} when the current became limited, we calculated $k_{\mathrm{O_2}}$ with (S11).

$$\dot{n}_{\mathrm{O_2,lim}} = k_{\mathrm{O_2}} \cdot \Delta C_{\mathrm{O_2}} = k_{\mathrm{O_2}} \cdot (C_{\mathrm{O_2,bulk}} - C_{\mathrm{O_2,Cat.}}) = k_{\mathrm{O_2}} \cdot C_{\mathrm{O_2,bulk}} \tag{S11}$$

We determined the bulk oxygen concentration, $C_{\mathrm{O_2,bulk}}$, with the ideal gas law (S12). We assumed the gas temperature was equal to the ambient temperature of $T = 20°\mathrm{C}$. The partial pressure of oxygen, $p_{\mathrm{O_2}}$, was calculated assuming a volumetric concentration of 21% of the recorded gas pressure, p_{G}, with (S13).

$$C_{\mathrm{O_2,bulk}} = \frac{p_{\mathrm{O_2}}}{R\,T} \tag{S12}$$

$$p_{\mathrm{O_2}} = 21\% \cdot p_{\mathrm{G}} \tag{S13}$$

Finally, the overall O_2 mass transfer coefficient of the GDE, $k_{\mathrm{O_2}}$ in cm s^{-1}, can be calculated with (S14) after substituting (S13) and (S12) into equation (S11) and rearranging the factors. The random error of the mass transfer

coefficient, $\sigma_{k_{O_2}}$, was also calculated using with (S14) by replacing the average $\bar{j}_{lim}$ with the average sample standard deviation of the limiting current density, $\bar{\sigma}_{j_{lim}}$.

$$k_{O_2} = \frac{c_{O_2,bulk}}{\dot{n}_{O_2,lim}} = \frac{21\% \cdot p_G \cdot F \cdot z}{R \cdot T \cdot \bar{j}_{lim}} \quad (S14)$$

The resulting overall mass transfer coefficients are displayed in **Figure S23** and listed in **Table S6**.

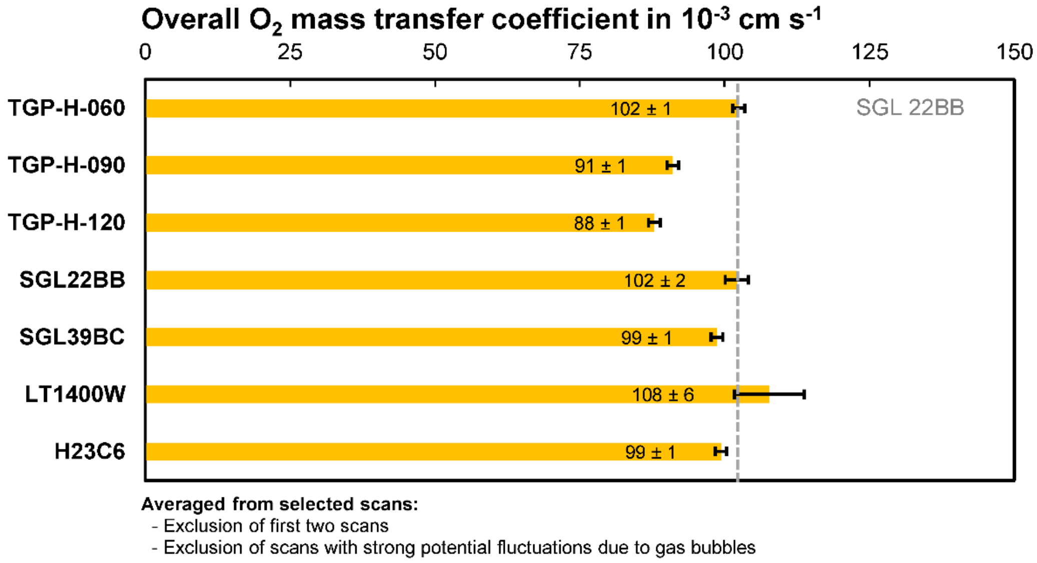

Figure S23: Overall O_2 mass transfer coefficients, k_{O_2}, determined with linear sweep voltammetry (LSV).

Table S6: Data processing overview for limiting overall O_2 mass transfer coefficients, k_{O_2}. The absolute pressure of the gas feed is p_G. The potential window of the limiting current density plateau is between the lower limit, $E_{lim,lower}$, and the upper limit, $E_{lim,upper}$. Scan numbers (#) used to determine average limiting current densities, $\bar{j}_{lim}$, and average sample standard deviations, $\bar{\sigma}_{j_{lim}}$. The limiting O_2 molar flux is $\dot{n}_{O_2,lim}$. The random error of the mass transfer coefficient is $\sigma_{k_{O_2}}$.

Material	Unit	TGP-H-060	TGP-H-090	TGP-H-120	SGL 22BB	SGL 39BC	LT1400W	H23C6
p_G	bar	1.290	1.533	1.242	1.344	1.370	1.280	1.264
$E_{lim,lower}$	V vs SHE	-1.75	-1.80	-1.45	-1.80	-1.60	-1.50	-1.45
$E_{lim,upper}$	V vs SHE	-1.30	-1.70	-1.15	-1.40	-1.20	-1.10	-1.20
Scan #	-	3, 4	3, 4, 5	3, 4, 5	3, 4	3, 5, 6, 7	3, 5	3, 4
$\bar{j}_{lim}$	mA cm^{-2}	440	464	363	456	450	459	418
$\bar{\sigma}_{j_{lim}}$	mA cm^{-2}	±5	±8	±10	±13	±9	±33	±5
$\dot{n}_{O_2,lim}$	10^{-6} mol cm^{-2} s^{-1}	1.14	1.20	0.94	1.18	1.17	1.19	1.08
$\boldsymbol{k_{O_2}}$	**cm s^{-1}**	**0.102**	**0.091**	**0.088**	**0.102**	**0.099**	**0.108**	**0.099**
$\boldsymbol{\sigma_{k_{O_2}}}$	**cm s^{-1}**	**±0.001**	**±0.001**	**±0.001**	**±0.002**	**±0.001**	**±0.006**	**±0.001**

Decomposition of overall O_2 mass transfer coefficient for SGL 22BB

We can decompose the overall mass transfer coefficient of SGL 22BB by using characterization from the fuel cell research of Reshetenko *et al.*.[17, 18] The overall mass transfer coefficient, k_i in cm s^{-1}, is a measure for the mass transfer rate from the bulk of the gas feed to the surface of the catalyst. It is the inverse of the overall resistance to mass transfer $R_{i,\mathrm{overall}}$ (S15).

$$\frac{1}{k_i} = R_{i,\mathrm{overall}} \tag{S15}$$

The overall resistance to mass transfer $R_{i,\mathrm{overall}}$ can be broken down the into mass transfer resistances present in the different domains of the GDE (S16).

$$R_{i,\mathrm{overall}} = R_{i,\mathrm{CFS}} + R_{i,\mathrm{MPL}} + R_{i,\mathrm{CL}} \tag{S16}$$

The components of the resistance can also expressed as an inverse of their mass transfer coefficients (S17).

$$\frac{1}{k_i} = \frac{1}{k_{i,\mathrm{m,CFS}}} + \frac{1}{k_{i,\mathrm{K,MPL}}} + \frac{1}{k_{i,\mathrm{K+film,CL}}} \tag{S17}$$

In the gas channel and the larger pores of the CFS, the transport takes place through molecular diffusion ($k_{i,\mathrm{m,CFS}}$). In the small pores of the MPL, the transport can take place through molecular and Knudsen diffusion ($k_{i,\mathrm{K,MPL}}$). Knudsen diffusion is a mechanism that dominates in small pores, in which the collisions of molecules with the pore walls are more frequent than the collisions of molecules with each other. In the CL, Knudsen diffusion takes place together with molecular diffusion through films of ionomer and electrolyte before reaching the catalyst surface ($k_{i,\mathrm{K+film,CL}}$).[17]

Using (S18) and (S19), we can decompose the experimentally determined $k_{\mathrm{O_2}}$ of our SGL 22BB sample with an estimate for $k_{\mathrm{O_2,m,CFS}}$ and $k_{\mathrm{O_2,K,MPL}}$.

$$R_{\mathrm{O_2,overall}} = R_{\mathrm{O_2,CFS}} + R_{\mathrm{O_2,MPL}} + R_{\mathrm{O_2,CL}} \tag{S18}$$

$$\frac{1}{k_{\mathrm{O_2}}} = \frac{1}{k_{\mathrm{O_2,m,CFS}}} + \frac{1}{k_{\mathrm{O_2,MPL}}} + \frac{1}{k_{\mathrm{O_2,K+film,CL}}} \tag{S19}$$

Reshetenko *et al*. performed an experimental study of GDL materials for polymer electrolyte fuel cells that provides useful information for this analysis. They were able to break down the overall mass transfer coefficient, $k_{\mathrm{O_2}}$, into mass transfer coefficients of the different GDE domains by using oxygen mixtures with different diluents.[17] Their study included the sample SGL 25BC, which is equivalent to the SGL 22BB of our study (**Table S7**).

Table S7: O_2 mass transfer coefficients recorded for SGL 25BC (equivalent to 22BB) taken from PEFC literature.[17] The measurements were carried out in N_2 media at a pressure of p_0 = 1.5 bar and a temperature of T_0 = 60°C. The overall O_2 mass transfer coefficient through the all domains of the GDE, $k_{\mathrm{O_2}}$, is broken down into the different domains k of the electrode (CFS, MPL, CL). The mass transfer resistances are given by the inverse of the individual mass transfer coefficients $R_k = 1/k_{\mathrm{O_2,k}}$ from Table 2 of Reshetenko *et al.*.[17]

Domain k	CFS	MPL	CL	GDE
Transport	molecular	Knudsen	Knudsen + film	overall
Symbol	$k_{\mathrm{O_2,m,CFS}}$	$k_{\mathrm{O_2,K,MPL}}$	$k_{\mathrm{O_2,K+film,CL}}$	$k_{\mathrm{O_2}}$
$\boldsymbol{k_{\mathrm{O_2},k}}$	2.82 cm s^{-1}	6.36 cm s^{-1}	1.65 cm s^{-1}	0.89 cm s^{-1}
$\boldsymbol{R_{i,k} = 1/k_{\mathrm{O_2},k}}$	0.35 s cm^{-1}	0.16 s cm^{-1}	0.61 s cm^{-1}	1.12 s cm^{-1}
$\boldsymbol{R_{i,k}/R_{i,\mathrm{overall}}}$	32%	14%	54%	100%

The data in **Table S7** show us that the mass transfer resistance in the CFS of SGL 25BC is 32% of the overall mass transfer resistance, $R_{\mathrm{O_2,overall}}$. We can adjust the mass transfer coefficients for the different conditions in the PEFC

(p_0 = 1.5 bar, T_0 = 60°C) to our conditions (p = 1.344 bar, T = 20°C) with equation (S20).[19] We replaced the binary diffusion coefficient $D_{k,i}$ of species k in (gas) medium i of this equation with the CFS mass transfer coefficient $k_{O_2,\mathrm{m,CFS}}$. This gives us an estimate of the mass transfer coefficient through the CFS $k_{O_2,\mathrm{m,CFS}}$ of our SGL 22BB sample (**Table S8**).

$$D_{k,i}(T,p) = D_{k,i,0}\left(\frac{T}{T_0}\right)^{1.5}\left(\frac{p_0}{p}\right) \tag{S20}$$

Because the Knudsen diffusion coefficient, $D_{O_2,\mathrm{K}}$, is proportional to the temperature, $T^{0.5}$,[19] we can adjust for the different temperature with (S21). The estimated Knudsen mass transfer coefficient for the MPL is given in **Table S8**.

$$k_{O_2,\mathrm{K,MPL}}(T,p) = k_{O_2,\mathrm{K,MPL}}\left(\frac{T}{T_0}\right)^{0.5} \tag{S21}$$

Table S8: Estimation of CFS mass transfer coefficient through CFS, $k_{O_2,\mathrm{m,CFS}}$, for 22BB based on the study of Reshetenko *et al.*.[17]

$k_{O_2,k}$	T	p_G	$k_{O_2,\mathrm{m,CFS}}$	Source / Comment
$k_{O_2,\mathrm{m,CFS}}$	60°C	1.5 bar	2.82 cm s^{-1}	PEFC literature from Table 2 of [17]
$k_{O_2,\mathrm{m,CFS}}$	20°C	1.334 bar	2.60 cm s^{-1}	Previous line adjusted with (S20)
$k_{O_2,\mathrm{K,MPL}}$	60°C		6.36 cm s^{-1}	PEFC literature from Table 2 of [17]
$k_{O_2,\mathrm{K,MPL}}$	20°C		5.97 cm s^{-1}	Previous line adjusted with (S21)

The contributions to the mass transfer resistance are then calculated with (S18) and (S19) (**Table S9**). We neglected the contribution of gas diffusion in the MPL. The resistance of CL makes up 94% of the total resistance of the GDE. The large mass transfer resistance of the CL is probably caused by the flooding of this layer with electrolyte.

Table S9: Estimation of mass transfer resistance contributions of CFS, MPL, and CL for 22BB at a pressure of p = 1.344 bar and a temperature of T = 20°C. We neglected molecular gas diffusion in the MPL.

Domain k	CFS	MPL	CL	GDE
Transport	molecular	Knudsen	Knudsen + film	overall
Symbol	$k_{O_2,\mathrm{m,CFS}}$	$k_{O_2,\mathrm{K,MPL}}$	$k_{O_2,\mathrm{K+film,CL}}$	k_{O_2}
$k_{O_2,k}$	2.60 cm s^{-1}	5.97 cm s^{-1}	0.108 cm s^{-1}	0.102 cm s^{-1}
$R_{i,k} = 1/k_{O_2,k}$	0.38 s cm^{-1}	0.17 s cm^{-1}	9.24 s cm^{-1}	9.79 s cm^{-1}
$R_{i,k}/R_{i,\mathrm{overall}}$	4%	2%	94%	100%

10. Stability test for CO_2 electrolysis

We carried out a stability test for the electrochemical CO_2 reduction for 20 h. We used a GDE with a SGL 39BC substrate, which was loaded with a catalyst layer of 1.27 mg Ag cm^{-2} and 20 wt% Nafion. As this study was a preliminary experiment, the experimental setup varied slightly from the process flow diagram shown in **Figure S16**:

- We carried out the experiments in the 2-compartment cell (**Figure S10**)
- Due to the larger electrode distance of this cell, our potentiostat was only able to reach 190 mA cm^{-2}
- Due to the membraneless configuration, the anode and cathode shared the same 1 M $KHCO_3$ electrolyte
- The electrolyte was supplied with a flow rate of 20 mL min^{-1} instead of 100 mL min^{-1}
- The CO_2 feed flow rate was 30 mLn min^{-1} instead of 50 mLn min^{-1}
- The CO_2 purge was 50 mLn min^{-1} instead of 80 mLn min^{-1}
- A polypropylene mesh was placed on the gas side of the GDE for mechanical support
- The flow rate of the product gas mixture was determined with a bubble flow meter

More detailed data for each GC injection are provided in the "Stability" sheet of the **Excel file**. The results of the stability test are summarized in **Figure S24**.

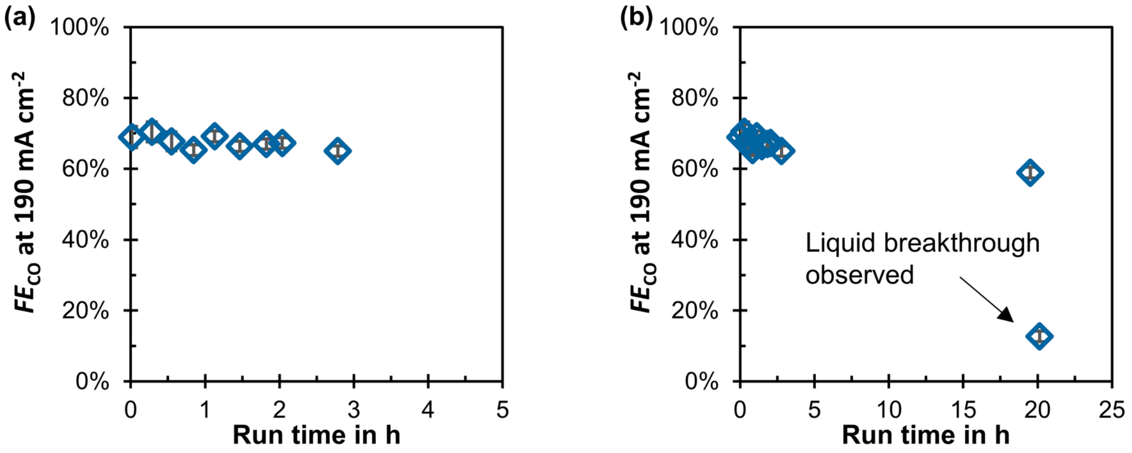

Figure S24: Results of stability test: Faradaic efficiency for CO at 190 mA cm^{-2} in flow-by mode. GDE material: SGL 39 BC coated with 1.27 mg Ag cm^{-2} and 20 wt% Nafion

The Faradaic efficiency for CO, FE_{CO}, remained stable at 68% ± 1.5% over the first 2 h of the stability test (**Figure S24 a**). We note that this is lower than the corresponding value we measured during our mass transfer characterization experiments: We measured a FE_{CO} of 75% ± 2.1% at a current density of 200 mA cm^{-2} for SGL 39BC (**Figure 7**). This lower performance is probably due to differences in the experimental setup for the stability test. Based on the sufficient short-term stability over 2 h, we can assume that no significant loss of performance occurred during the short collection period (10 min) of the FE_{CO} data, which we used to compare the different GDL materials at 200 mA cm^{-2} (**Figure 7**).

The FE_{CO} declined slowly to a value of 59% ± 1.4% over a run time of 19.5 h (**Figure S24 b**). After 20 h, we suddenly observed flooding of the GDE and liquid breakthrough. This loss of stability could be explained by the following mechanisms (or a combination of both):

- Carbonate formation in the GDE:[13]
    - Electrolyte evaporates and increases the local salt concentration
    - Carbonate salts (K_2CO_3 or $KHCO_3$) precipitate in the pores of the GDE
    - The salt crystallites decrease the hydrophobicity of the pore surface
    - The capillary pressures of the pores are reduced and flooding occurs
- (Electro-)chemical degradation of the GDE:[20, 21]
    - The very negative cathode potential (–1.9 V vs SHE) and/or the high local pH in the CL degrade the carbon or even the PTFE of the GDL
    - The degraded carbon and/or PTFE has a lower hydrophobicity
    - The capillary pressures of the pores are reduced and flooding occurs

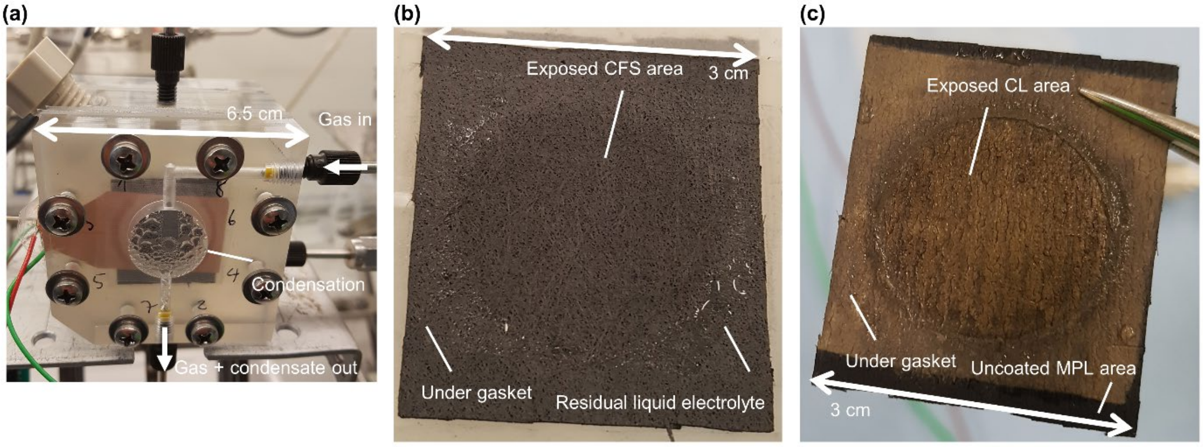

Figure S25: Pictures of stability test: **(a)** Condensation during stability test **(b)** Picture of CFS after 20 h stability test **(c)** Picture of CL after 20 h stability test.

We observed the continuous formation of condensation on the outer wall of the gas compartment during the stability test (**Figure S25 a**). This observation makes the loss of hydrophobicity due to gradual carbonate formation in the GDE a plausible hypothesis. We suspect that the heat produced through the electrochemical reactions in the CL lead to an evaporation of water from the electrolyte. The water vapor must have then diffused through the GDE and oversaturated the already humidified gas stream in the gas compartment. The condensation occurred at the outer wall because this is the coldest point in the gas compartment.

To test the hypothesis of carbonate salt formation, we disassembled the cell and inspected the CFS side (**Figure S25 b**) and the CL side of the GDE (**Figure S25 c**). The CFS appeared relatively dry and there were no salt deposits visible on the surface. Carbonate salt formation, however, could still be a possible degradation mechanism because the precipitation might have occurred inside the pores and not be visible from the outside.

To test the hypothesis of (electro-)chemical degradation, we measured the change in the static contact angle. We rinsed the spent sample with isopropyl alcohol to remove any residual electrolyte and let it dry in air. Our results show that the contact angle of the CFS decreased significantly due to the 20 h stability test at 190 mA cm^{-2} (**Table S10**). We can, therefore, assume that the (electro-)chemical degradation significantly limits the stability of carbon-based GDEs. The effects of this degradation mechanism on the surface chemistry should be investigated more systematically in a future study. It might be possible to mitigate this degradation mechanism by reducing the cathode overpotential through improved catalyst materials and lower mass transfer resistance in the CL.

Table S10: Contact angle change after stability test with SGL 39 BC. CO_2 electrolysis was conducted for 20 h at 190 mA cm^{-2}. Only one measurement was possible for the MPL of the spend sample because the uncoated area was limited in size.

Contact angle	Fresh sample	Spent sample
**CFS**	149 ± 1.0°	128 ± 1.2°
**MPL**	153 ± 0.8°	133° (only one data point)
**CL**	123 ± 1.4°	109 ± 5.4°

References

1. Forner-Cuenca, A., E.E. Penn, A.M. Oliveira, and F.R. Brushett, *Exploring the Role of Electrode Microstructure on the Performance of Non-Aqueous Redox Flow Batteries.* Journal of The Electrochemical Society, 2019. **166**(10): p. A2230.
2. Endrődi, B., G. Bencsik, F. Darvas, R. Jones, K. Rajeshwar, and C. Janáky, *Continuous-Flow Electroreduction of Carbon Dioxide.* Progress in Energy and Combustion Science, 2017. **62**: p. 133-154.
3. Duarte, M., B. De Mot, J. Hereijgers, and T. Breugelmans, *Electrochemical Reduction of CO_2: Effect of Convective CO_2 Supply in Gas Diffusion Electrodes.* ChemElectroChem, 2019. **6**(22): p. 5596-5602.
4. Weber, A.Z., R.M. Darling, and J. Newman, *Modeling Two-Phase Behavior in PEFCs.* Journal of the Electrochemical Society, 2004. **151**(10): p. A1715-A1727.
5. De Gennes, P.-G., F. Brochard-Wyart, and D. Quéré, *Capillarity and Wetting Phenomena: Drops, Bubbles, Pearls, Waves*. 2013: Springer Science & Business Media.

6. Gostick, J.T., M.W. Fowler, M.A. Ioannidis, M.D. Pritzker, Y.M. Volfkovich, and A. Sakars, *Capillary Pressure and Hydrophilic Porosity in Gas Diffusion Layers for Polymer Electrolyte Fuel Cells.* Journal of power sources, 2006. **156**(2): p. 375-387.
7. Gurau, V., M.J. Bluemle, E.S. De Castro, Y.-M. Tsou, J.A. Mann, and T.A. Zawodzinski, *Characterization of Transport Properties in Gas Diffusion Layers for Proton Exchange Membrane Fuel Cells: 1. Wettability (Internal Contact Angle to Water and Surface Energy of Gdl Fibers).* Journal of Power Sources, 2006. **160**(2): p. 1156-1162.
8. Friess, B.R. and M. Hoorfar, *Measurement of Internal Wettability of Gas Diffusion Porous Media of Proton Exchange Membrane Fuel Cells.* Journal of Power Sources, 2010. **195**(15): p. 4736-4742.
9. Wood, D.L., C. Rulison, and R.L. Borup, *Surface Properties of Pemfc Gas Diffusion Layers.* Journal of The Electrochemical Society, 2010. **157**(2): p. B195.
10. Abbou, S., K. Tajiri, E. Medici, and J.S. Allen, *Characterization of Water Transport in PEMFC Electrode Using the Washburn Method.* ECS Transactions, 2017. **80**(8): p. 87-93.
11. Mortazavi, M. and K. Tajiri. *Impact of Gas Diffusion Layer Properties on Liquid Water Breakthrough Pressure in Polymer Electrolyte Fuel Cell*. in *ASME 2013 11th International Conference on Fuel Cell Science, Engineering and Technology collocated with the ASME 2013 Heat Transfer Summer Conference and the ASME 2013 7th International Conference on Energy Sustainability*. 2013. American Society of Mechanical Engineers Digital Collection.
12. Mortazavi, M. and K. Tajiri, *In-Plane Microstructure of Gas Diffusion Layers with Different Properties for PEFC.* Journal of fuel cell science and technology, 2014. **11**(2).
13. Leonard, M.E., L.E. Clarke, A. Forner-Cuenca, S.M. Brown, and F.R. Brushett, *Investigating Electrode Flooding in a Flowing Electrolyte, Gas-Fed Carbon Dioxide Electrolyzer.* ChemSusChem, 2019. **13**(2): p. 400-411.
14. Mortazavi, M. and K. Tajiri, *Liquid Water Breakthrough Pressure through Gas Diffusion Layer of Proton Exchange Membrane Fuel Cell.* International Journal of Hydrogen Energy, 2014. **39**(17): p. 9409-9419.
15. Zeng, Z. and R. Grigg, *A Criterion for Non-Darcy Flow in Porous Media.* Transport in Porous Media, 2006. **63**(1): p. 57-69.
16. Hori, Y.i., *Electrochemical CO_2 Reduction on Metal Electrodes*, in *Modern Aspects of Electrochemistry*. 2008, Springer. p. 89-189.
17. Reshetenko, T. and B.L. Ben, *Impact of a Gas Diffusion Layer's Structural and Textural Properties on Oxygen Mass Transport Resistance in the Cathode and Performance of Proton Exchange Membrane Fuel Cells.* Electrochimica Acta, 2021. **371**: p. 137752.
18. Reshetenko, T.V. and J. St-Pierre, *Separation Method for Oxygen Mass Transport Coefficient in Gas and Ionomer Phases in Pemfc Gde.* Journal of The Electrochemical Society, 2014. **161**(10): p. F1089-F1100.
19. Wang, C.-Y., *Fundamental Models for Fuel Cell Engineering.* Chemical Reviews, 2004. **104**(10): p. 4727-4766.
20. Yang, K., R. Kas, W.A. Smith, and T. Burdyny, *Role of the Carbon-Based Gas Diffusion Layer on Flooding in a Gas Diffusion Electrode Cell for Electrochemical CO_2 Reduction.* ACS Energy Letters, 2021. **6**(1): p. 33-40.
21. Shapoval, G., A. Tomilov, A. Pud, and V. Vonsyatskii, *Electrochemical Reductive Destruction of Polytetrafluoroethylene.* Theoretical and Experimental Chemistry, 1984. **20**(2): p. 234-236.
